# MSV: a modular structural variant caller that reveals nested and complex rearrangements by unifying breakends inferred directly from reads

**DOI:** 10.1186/s13059-023-03009-5

**Published:** 2023-07-17

**Authors:** Markus Schmidt, Arne Kutzner

**Affiliations:** 1 Biomedical Center Munich, Department of Physiological Chemistry, Ludwig-Maximilians-Universität, Großhaderner Str. 9, 82152 Planegg-Martinsried, Germany; 2 Department of Information Systems, College of Engineering, Hanyang University, 222 Wangsimni-Ro, Seongdong-Gu, Seoul, 133-791 Republic of Korea

**Keywords:** Nested SV, Complex SV, Alignment, MEMs, Seeds, Pan-genomes, Ambiguity

## Abstract

Structural variant (SV) calling belongs to the standard tools of modern bioinformatics for identifying and describing alterations in genomes. Initially, this work presents several complex genomic rearrangements that reveal conceptual ambiguities inherent to the representation via basic SV. We contextualize these ambiguities theoretically as well as practically and propose a graph-based approach for resolving them. For various yeast genomes, we practically compute adjacency matrices of our graph model and demonstrate that they provide highly accurate descriptions of one genome in terms of another. An open-source prototype implementation of our approach is available under the MIT license at https://github.com/ITBE-Lab/MA.

**Supplementary Information:**

The online version contains supplementary material available at 10.1186/s13059-023-03009-5.

## Background

Structural variant (SV) calling is a popular technique for discovering and describing genomic differences and rearrangements. Examples of the application of SV calling are phylogenetic analyses and the discovery of genetic disorders. SV callers can be classified as alignment-based, assembly-based, and meta-callers. Alignment-based callers rely on short reads (Illumina) or long reads (PacBio, Oxford Nanopore) [1–4]. Here SV callers exploit one or several types of evidence for SV detection such as (1) deviations from the expected distance between paired reads (read-pair-based calling), (2) the locations of supplementary alignments for chimeric reads (split-read-based calling), and (3) regions with particularly high or low coverage on the reference genome regarding alignments. Assembly-based callers detect SV by comparing assemblies, e.g., in [5–8]. Finally, meta-callers [9–11] try to achieve high accuracy and high recall rates by combining the output of other SV callers. Overviews presenting all these types of SV callers together with their characteristics and their respective performances can be found in [12–15].

Our work starts by reporting shortcomings of state-of-the-art read-pair and split-read-based SV calling. Here we identify ambiguities inherent to the description of genomic rearrangements via basic SV [16]. As a solution, we introduce a description of genomic rearrangements using skew-symmetric graphs. A highlight of our graph model is a folding scheme for adjacency matrices that unifies forward strand and reverse strand. The general suitability of graphs for the representation of genomic rearrangements is already demonstrated in [17, 18]. Furthermore, we inspect the negative side effects of alignments on SV calling that result from their path-oriented nature. For circumventing these side effects, our skew-symmetric graphs are computed using seeds merely. This seeding is extended by a recursive reseeding technique that takes the position of Dynamic Programming [19, 20] usually incorporated with alignment computations (e.g., in the aligners Minimap2 [21] and MA [22]). A practical evaluation using the two *Saccharomyces paradoxus* (wild yeast) genomes UFRJ50816 and YPS138 (data published by [23]) shows the viability of our approach. In this context, we express the genome UFRJ50816 in terms of the genome YPS138 via a graph that is computed either from the assemblies of both genomes or from PacBio reads and Illumina reads. A discussion of our approach emphasizes its suitability with graph genomes and, in this way, with pan genomes.

## Results

### Ambiguities inherent to the description of genomic rearrangements via basic SV

**Fig. 1:**
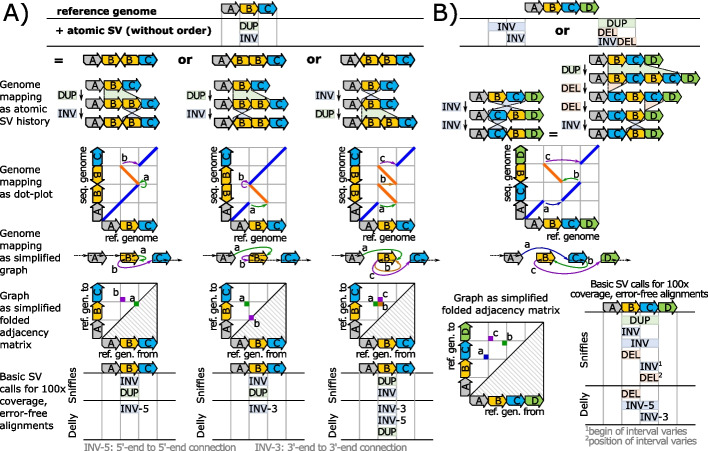
Examples of ambiguities resulting from the description of genomic rearrangements via basic SV. **A** shows a situation, where a duplication and an inversion correspond to three different outcomes, which are depicted by diagrammatic dot-plots (for a description of dot-plots and their diagrammatic representation see Additional file 2). Each outcome can be uniquely represented by a graph and its folded adjacency matrix. Here we show simplified versions of the graphs and their folded adjacency matrices merely. The unfolded matrices as well as the full graphs for all examples are given in Additional file 3. For testing SV callers that report genomic rearrangements via basic SV, such as Sniffles and Delly, we simulate 100 × coverage of error-free alignments for all cases with a read length of 1000 nt for long reads and 250 nt for paired-end short reads. **B** displays an example, where different rearrangements via basic SVs lead to equal outcomes. Here, a pair of inversions is equal to a duplication followed by two deletions and an inversion. As for **A**, the corresponding graph, folded adjacency matrix, and output of real-world SV callers are shown for both examples. Additional file 3 gives an analogous example, where a duplication followed by two inversions leads to the same outcome as two duplications followed by one inversion

In the following, we assume that genomic rearrangements are represented by combinations of basic SV, where a basic SV is one of the following four elementary reorganizations of the reference genome: Deletion, Insertion, Inversion or Duplication. Some state-of-the-art SV callers [1, 4] attempt to represent genomic rearrangements by nesting these basic SV. However, this representation scheme comprises inherent ambiguities in two ways: (1) a set of basic SVs can describe multiple reorganizations and, on the contrary, (2) a single genomic rearrangement can be described by multiple sets of basic SVs. Figure 1 visualizes these ambiguities via examples. In Fig. 1A, the combination of a duplication and an inversion describes three different genomic outcomes. In Fig. 1B, a reference $ABCD$ is reorganized to $AC\widetilde{B}D$ either with two inversions or with a duplication followed by two deletions and an inversion. Recently published works are aware of these ambiguities [24–27]. As an intermediate solution, they exclude complex genomic rearrangements that pose unmanageable ambiguities to them.

The abovementioned ambiguities can be eliminated by expressing the outcome of the rearrangement process (instead of the process itself) in terms of the reference. This can be accomplished by, e.g., dot-plots as shown in Fig. 1. Each dot-plot, in turn, can be translated into a genome-mapping graph, where all breakend pairs of the dot-plot become edges. (The notion breakend is similar to the notion breakpoint as explained in Additional file 1). Moreover, the genome-mapping graph can be represented using an adjacency matrix that can be efficiently stored in a database, a CSV file, or by tying together breakends in the VCF format [28] (using the BND tag and no other tag). For unifying forward and reverse strands, an adjacency matrix can be folded. A detailed definition of our graph-model, the inference of adjacency matrices, and their folding scheme is given in the methods section.

We now analyze the practical relevance of the abovementioned ambiguities by inspecting the output of the popular SV callers Sniffles [29] and Delly [4]. For this purpose, for each dot-plot, we generate a set of error-free alignments, which reflects the rearrangement of the respective dot-plot and fully covers the *y*-axis. A detailed description of the experimental setup is given in Additional files 4 and 5. We generate 100 × coverage and do not induce any noise, e.g., sequencer errors, into the alignments. This exclusion of noise is for avoiding additional complications for SV callers besides the nested variants themselves. The generated queries are forwarded to the SV callers for evaluation and their output is visualized in the bottom row of Fig. 1. All SV callers reconstruct the reference positions of the breakends successfully but struggle with the abovementioned ambiguities. For subfigure A, Sniffles reports an inversion and duplication for all three reorganizations. Therefore, Sniffles recognizes the basic SV correctly but cannot distinguish between the three cases. Delly distinguishes all three cases but does not report the duplication in the first two rearrangements. For Fig. 1B, Sniffles returns a combination of both development histories, where the basic SV are sorted by their start position on the reference (due to this sorting both histories appear intermingled). Delly’s output seems to comprise fragments of both development histories. Using our proposed representation scheme, all cases can be represented unambiguously by a unique adjacency matrix. Furthermore, there are SV callers, such as Gridss [30] and Manta [3], which avoid basic SV calls for reporting variants and make use of a BND-call representation of the VCF format [28] instead. As a consequence, these callers do not struggle with the ambiguities reported here. We examine the equivalence of the BND-call representation, our adjacency matrix approach, and a dot-plot representation in the discussion section.

### Alignments can conceal SV

**Fig. 2:**
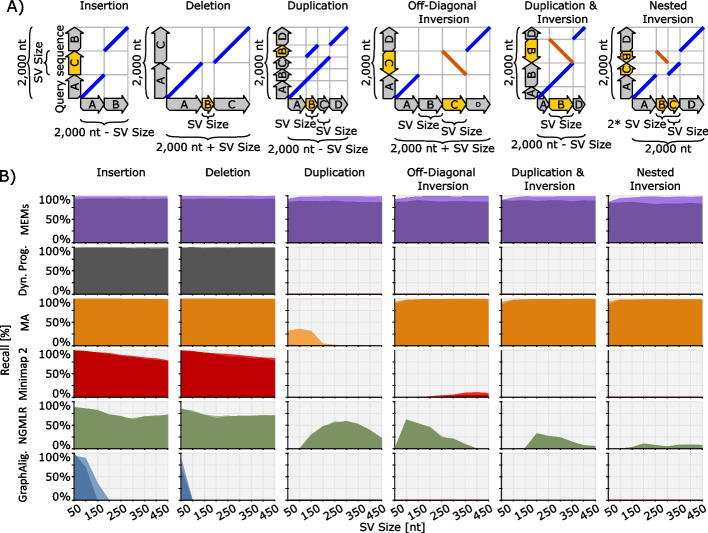
The figure compares Dynamic Programming (gray), MEMs (black), MA (orange), Minimap2 (red), NGMLR (green), and GraphAligner (blue) regarding the rediscovery of several types of SV. The human genome GRCh38.p12 is used as the reference genome. Subfigure **A** visualizes the analyzed genomic rearrangements using diagrammatic dot-plots. Blue lines indicate matches on the same strand, while orange lines represent matches on opposite strands. A description of diagrammatic dot-plots can be found in Additional file 2. Subfigure **B** displays the rediscovery rate as a function of the SV size (see subfigure **A**) for the various kinds of SV in **A**. Each diagram shows two curves. Here, each point of a curve represents an average measurement for 1000 query sequences. The lighter-colored areas show recall rates for idealized error-free queries, while the darker areas report rates for simulated PacBio CCS reads. The diagrams for Dynamic Programming show rediscovery rates for banded global Dynamic Programming with two-piece affine gap costs. Here, the global Dynamic Programming is merely applied to the reference section that a query originates from

SV callers recognize genomic rearrangements using the locations of their breakends. Here many state-of-the-art SV callers [1, 13, 14] rely on alignments of split-reads (chimeric reads). Therefore, these SV callers require their aligner to deliver precise and consistent breakend locations. Aligners are designed to find maximally scored paths in the tradition of Needleman-Wunsch’s (NW) [19] and Smith-Waterman’s [20] algorithms. Such a path-based approach is well-suited for the discovery of mutations, short insertions, and short deletions. However, long insertions and long deletions oppose maximally scored paths. This problem is well known and reflected by the introduction of two-piece affine gap-costs [31]. Furthermore, the path-driven approach contradicts duplications and inversions as indicated by the Dynamic Programming (DP) curves in Fig. 2B. State-of-the-art aligners attempt to compensate for these shortcomings by identifying special cases using tailored strategies and reporting them via supplementary alignments. For example, Minimap2 tries to identify potential inversions using a z-drop heuristic during DP and verifies these potential inversions via DP on the reverse complement.

In Fig. 2, we evaluate the aligners Minimap2 [21], NGMLR [29], GraphAligner [32], and MA [22] in the context of the abovementioned shortcomings (Additional file 5 lists the versions of all software used for the analysis). There, we benchmark the discovery rates of the aligners for the following SV types: (1) a plain deletion; (2) a plain insertion; (3) a duplication, where a gap occurs between the original and copied segment; (4) a deletion directly followed by an inversion; (5) an inverted duplication (identical to the leftmost case in Fig. 1A); and (6) two nested inversions (identical to the case in Fig. 1B. In all six cases, the aligner’s query sequences span over the complete SV. Each query is generated by performing the following three steps: (1) We pick a randomly located interval of a given size (this size is denoted on the *x*-axis of the respective case in Fig. 2A on the human genome assembly GRCh38.p12). (2) The sequence in that interval is decomposed into sections as visualized on the *x*-axis (sections named $A$, $B$, $C$, …). (3) The query is generated by copying and rearranging these sections as shown on the *y*-axis. All queries generated by the above scheme are of equal size (2000 nt). We generate 1000 queries for each SV size of each SV-variant, where each query is generated from a different, randomly chosen reference interval. Following the generation, the query sequences are forwarded to the aligner. Using the CIGARs of the primary alignments and the supplementary alignments, we verify an aligner’s discovery of all breakends that belong to the respective SV. Here, we grant a tolerance of 25 nt and verify reference locations as well as query locations of breakends. An SV counts as rediscovered if all its breakends are found. To account for sequencing errors, we additionally simulate substitutions, insertions, and deletions on queries with error rates that mimic PacBio HiFi reads, using the simulator published in [22]. Additional file 6 details the exact error simulation approach. As visible in Fig. 2B, erroneous reads have a limited impact on the recall rates of aligners and do not affect the range of SV types that are concealed by them.

In Additional file 6, we repeat the experiment of Fig. 2 with the query sizes 1000 nt and 20,000 nt. There, recall rates show improvements with increased query size, particularly for NGMLR [1]. However, the general shape of the curves stays unaltered since the size of the reorganized segments does not change.

Figure 2B indicates the limited success of state-of-the-art aligners in overcoming their path-oriented design. Aligners recognize some cases comprising duplications and inversions while failing on others. Maximal Exact Matches (MEMs) are a particular form of seeds, where seeds are equivalences between a reference genome and a read, typically used by an aligner as the basis for alignment computation. Informally, a MEM is such an equivalence between two sequences (e.g., reference genome and read) that cannot be extended in either direction without encountering a mismatch or the outer end of one (or both) of the sequences. A formal definition of MEMs can be found in [22]. The MEM’s curves, in contrast to the aligner’s curves, illustrate that this type of seed is sufficient for recognizing all analyzed cases. For MEMs, the impact of erroneous reads on the recall rates is higher than for aligners or DP, but still moderate. This can be attributed to the fact that, in the presence of sequencer errors, MEMs may not extend to the exact location of a breakend, whereas aligners accurately recover exact breakends by using DP. In our SV calling approach, we address imprecise breakends by employing techniques such as reseeding, fuzzy edges, and a statistical approximation of true entry locations. These techniques are explained in detail in the methods section.

The failing of aligners can be explained by their path-driven selection of seeds that occurs as part of the seed-processing step (e.g., chaining), where aligners purge relevant seeds. While small deletions and insertions are well discovered by all aligners, their discovery rates decrease with increasing indel size. This is in accordance with the abovementioned opposition of the path-oriented scoring scheme to long indels. Since the MEM and DP curves in Fig. 2B indicate that seeds and two-piece affine gap-costs cope well with larger indels, the worse behavior of all aligners for indels must again originate from their seed-processing step (e.g., chaining [33]).

Aligners rely on “occurrence filtering” for coping with the ambiguity of genomes. For keeping fairness, we mimic this occurrence filtering during MEM computation by discarding all MEMs that surpass a given threshold of occurrences on the genome. Except for insertions and deletions, Fig. 2B shows a slightly decreasing discovery rate with MEMs for small SV-sizes. This can be explained by the mimicked filtering in combination with the ambiguity of the human genome because small seeds tend to be purged by occurrence filtering and so the corresponding breakends cannot be discovered anymore. As with alignments, a breakend is considered as “discovered by a MEM” if one of the MEMs endpoints and the breakend have equal read and reference positions. Furthermore, an SV counts as rediscovered by MEMs if all its breakends are found. Please note that all cases investigated in Fig. 2 mimic real-world rearrangements that we encounter during our analysis of the yeast genomes UFRJ50816 and YPS138.

Some aligners, e.g., Minimap2, can be configured to report all discovered chains via supplementary alignments. If configured this way, an aligner’s recall rate in the above analysis increases significantly (as shown in Additional file 6 for Minimap2). However, the configuration results in an abundance of CIGARS without any contextual information among them. Without this contextual information, the CIGARs are of similar value as pure MEMs in the context of SV calling.

### SV calling using genome-mapping graphs inferred from MEMs

**Fig. 3:**
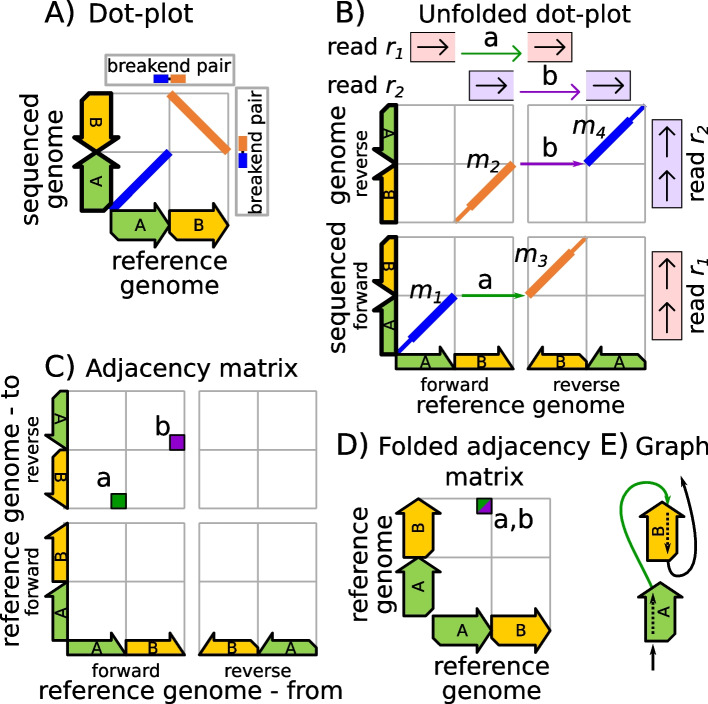
The figure displays the basic design of our approach for SV calling using an example. **A** The dot-plot diagrammatically visualizes an inversion of section ‘B’ on the sequenced genome. This inversion induces one breakend pair on the reference genome and one breakend pair on the sequenced genome. **B** shows the dot-plot of **A** unfolded into the forward strand and reverse strand. There are two reads ${r}_{1}$ and ${r}_{2}$, where ${r}_{1}$ originates from the forward strand and ${r}_{2}$ originates from the reverse strand of the sequenced genome. The locations of both reads on the sequenced genome (right) and the reference genome (top) are visualized, where the reads decompose into two segments on the reference genome. The reads indicate the inversion’s breakend pairs, once on the forward strand via the arrow labeled “a” and once on the reverse strand via the arrow labeled “b.” **C** The inversion’s breakend pairs are visualized in a corresponding adjacency matrix via the matrix entries labeled “a” and “b.” The *x*-position of an entry (breakend pair) is the origin position on the reference, while the *y*-position corresponds to the destination position. **D** We apply our matrix folding scheme to the adjacency matrix of subfigure **C** for unifying breakend-pair calls (matrix entries) from forward and reverse strand reads. **E** shows the genome mapping graph defined by the adjacency matrices of **C** and **D**

We now outline our technique for computing genome-mapping graphs from MEMs. Using the inversion shown in Fig. 3A as an example, we explain our approach step-by-step. First, we unfold the reference genome and represent it as a string of nucleotides so that the forward strand is directly followed by the reverse strand. Using this unfolded reference genome together with a set of given reads, we compute a set of MEMs and record the linkage between the MEMs’ breakends during computation. Figure 3B shows the computation of four MEMs ${m}_{1}$ to ${m}_{4}$ for the two exemplary reads ${r}_{1}$ and ${r}_{2}$ that are assumed to be error-free for simplicity. The four MEMs are connected at their breakends via the two links labeled “a” (end of ${m}_{1}$ to begin of ${m}_{3}$) and “b” (end of ${m}_{2}$ to begin of ${m}_{4}$). Next, the linkage between MEMs is mapped into an adjacency matrix that runs along the reference genome on both axes. Informally, this adjacency matrix expresses the sequenced genome in terms of the reference genome via neighborhood relationships, i.e. the matrix indicates which nucleotides of the reference genome appear as neighbors on the sequenced genome. The matrix can be simplified by omitting entries that do not indicate differences between reference genome and sequenced genome (such entries appear directly over the matrix diagonal as described in the methods section). The neighborhood relationships are expressed in the matrix by indicating ‘from’ which position on the reference genome (*x*-axis position of a matrix entry) we have to go “to” which position on the reference genome (*y*-axis position of a matrix entry) for reconstructing the sequenced genome. For example, in Fig. 3B the arrow of the breakend-pair labeled “a” originates from the last nucleotide of the sequence ‘A’. Therefore, the matrix entry “a” in Fig. 3C is placed above the end of the sequence ‘A’ on the forward strand. Accordingly, the matrix entry’s vertical position is defined by the arrow’s end, which points to the first nucleotide of the sequence ‘B’ on the reverse strand. For inferring the adjacency matrix position of a breakend pair, we merely require the immediate surrounding area on the sequenced genome, which can be provided by a sequencing read. As opposed to the dot-plots in Fig. 3A and B, both axes of the adjacency matrix run along the reference genome. This is vital, as the sequenced genome is unknown during SV calling. The matrix shown in Fig. 3C is not suitable for describing structural variations because it comprises the example’s inversion twice. To solve this problem, the matrix is folded in 3 steps (the folding scheme is explained in the methods section) and reduced to the matrix shown in Fig. 3D. Here the two entries “a” and “b” of the unfolded matrix are unified to a single entry “a/b” that describes the example’s inversion uniquely. Such a folded matrix can then be visualized as a genome-mapping graph that expresses the sequenced genome in terms of the reference genome. Figure 3E shows this graph for the example. In the absence of ambiguities induced by genomic repetitions, the genome-mapping graph allows a directed reconstruction of the sequenced genome from the reference genome. In the case of ambiguities (e.g., in the case of duplications, where the reconstruction runs into a cycle)*,* an additional graph traversal is required that decides the order of entry visits in ambiguous situations. A detailed description of our SV calling approach is given in the methods section. This includes the proposal of a memory-efficient representation of our adjacency matrices.

### Incorporating genome repetitiveness and sequencer errors into SV calling

**Fig. 4:**
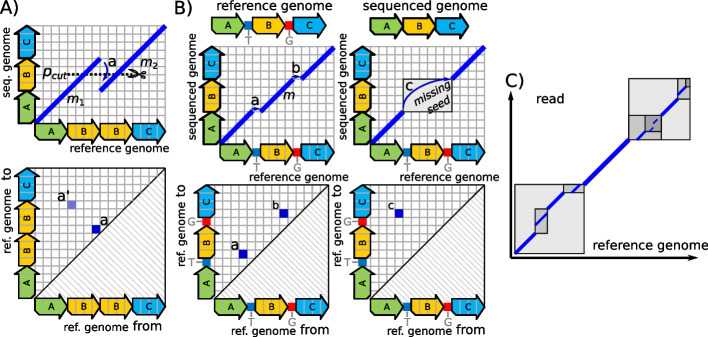
**A** shows a problem caused by the repetitiveness of the reference genome. In the sequenced genome, one of the occurrences of $B$ is missing. The two seeds ${m}_{1}$ and ${m}_{2}$ that overlap on the *y*-axis (top) corresponds to the erroneous entry $a$ (bottom). Our overlap-elimination technique (as described in the methods section) resolves this problem by shortening ${m}_{1}$ and ${m}_{2}$ to the line ${p}_{cut}$. The result of this shortening is the correct entry $a{\prime}$. **B** Two 1 nt deletions between the reference genome and sequenced genome create the small seed $m$ (top-left). $m$ is assumed to be deleted by the occurrence filtering of our approach (top-right). This deletion leads to the replacement of the two correct entries $a$ and $b$ (bottom-left) with a wrong entry $c$ (bottom-right). For salvaging such lost seeds, we rediscover $m$ via a reseeding within the gray boxed area (top-right). **C** visualizes our reseeding technique for a larger area, where each box corresponds to a recursive call and the gray’s darkness indicates the recursion depth

Repeats on the reference genome can cause MEMs to overlap on the *y*-axis (on the read) as shown in Fig. 4A. There, the MEMs ${m}_{1}$ and ${m}_{2}$ both cover the section labeled $B$ on the *y*-axis. This overlap leads to the erroneous matrix entry labeled $a$. This entry expresses that, on the sequenced genome, the reference’s first instance of $B$ is followed by the reference’s second instance of $B$. However, for correctly representing the sequenced genome via the adjacency matrix, we instead express that there exists only one instance of $B$ on the sequenced genome. A graph genome, where such repetitiveness is modeled by edges instead of vertices, would eliminate this problem (more details on graph genomes can be found in the discussion section). In the methods section, we introduce an algorithmic scheme that shortens overlapping seeds for resolving such overlaps on sequential reference genomes.

The repetitiveness of genomes creates further issues. In the following, let $S$ be a sequence that occurs once on the reference genome but $n$ times on the sequenced genome. This leads to a situation, where the vertex belonging to the first or last nucleotide of $S$ comprises $n$ inbound or outbound edges, respectively. Relations between such inbound and outbound edges cannot be expressed by the graph itself. Instead, they must be defined by a graph traversal. Since our approach currently does not compute such a traversal, we avoid these regions by applying a filtering approach on all reads as described in the methods section. Now, let $S$ be a sequence that occurs $n$ times on the reference genome and at least once on the sequenced genome. In this case, an occurrence of $S$ on a read triggers $n$ matches on the reference, where all of them are false positives except for one. For handling this situation, we discard all seeds. This technique is similar to the “occurrence filtering” that is part of the seeding step of many aligners [21, 22, 34]. The discarding of ambiguous seeds primarily removes small seeds since the number of occurrences of a seed on a genome is expected to increase with decreasing size of the seed. However, small seeds are required if two differences between read and reference are close to each other. Such differences can be caused by genomic rearrangements, sequencer errors, or combinations of them. If the seed between two differences is missing, two correct entries in the adjacency matrix are replaced by a single wrong entry (see Fig. 4B). For tackling this problem, we propose an efficient reseeding scheme for the retrieval of small seeds on a locally confined region of the reference (see Fig. 4C). The required basic techniques for obtaining such seeds using an index computed on the fly are proposed in [35]. There, a hash table is used for computing Minimizers that are merged and extended afterward for obtaining MEMs. For each MEM, we search for smaller undiscovered MEMs occurring immediately before and after it. This process is recursively extended to freshly discovered MEMs until they are expected to be too small (down to 5nt). The detailed process is described in the Methods section.

### Evaluation using yeast genomes

So far, we analyzed individual aspects of our SV calling approach merely. Now, we perform a comprehensive evaluation using the real-world data proposed in [23] for *Saccharomyces paradoxus* (wild yeast). We work with yeast genomes due to their small size and moderate repetitiveness in comparison to, e.g., the human genome. In the Discussion section, we outline extensions and modifications of our approach that would allow its application to the human genome. The data in [23] are particularly well suited for our analysis because they comprise independently assembled genomes for several strains of wild and domestic yeast, which can be used as ground truth for our analysis. We focus on the genomes UFRJ50816 and YPS138 here. These genomes comprise complex genomic rearrangements (e.g., six clustered inversions, some of which are overlapping) that are similar to the situations shown in Fig. 1B2. Finally, the yeast genomes in [23] are all sequenced in their haploid or homozygous diploid forms, which simplifies SV calling compared to diploid genomes comprising heterozygous loci.

Due to the reported ambiguities of basic SV, an evaluation of our approach using VCF-formatted data as ground truth is not feasible. For overcoming this problem, we compute a set of adjacency matrix entries based on a reference assembly (we use YPS138) and a sequenced assembly (we use UFRJ50816) as ground truth entries. The entries are inferred from a comprehensive set of MEMs for the assemblies. Here, we rely on the same algorithmic techniques for computing seeds and matrix entries as we use for reads (see methods section). The overall scheme for the computation of ground-truth entries is given in Additional file 7. For the $\sim$ 12Mio bp long yeast genomes, this ground-truth matrix comprises 45,533 entries. In the following, it is denoted by ${M}_{T}$.

The theoretic foundations of our approach demand that the sequenced assembly corresponds to one specific traversal throughout the genome-mapping graph, where the graph is defined by the ground-truth entries and the traversal is determined by a visiting order among the entries. For UFRJ50816 and YPS138 we could fully reconstruct the sequenced genome using a prototype implementation, which is described in Additional file 8. This, in turn, proves the correctness of the practically computed ground-truth entries.

**Fig. 5:**
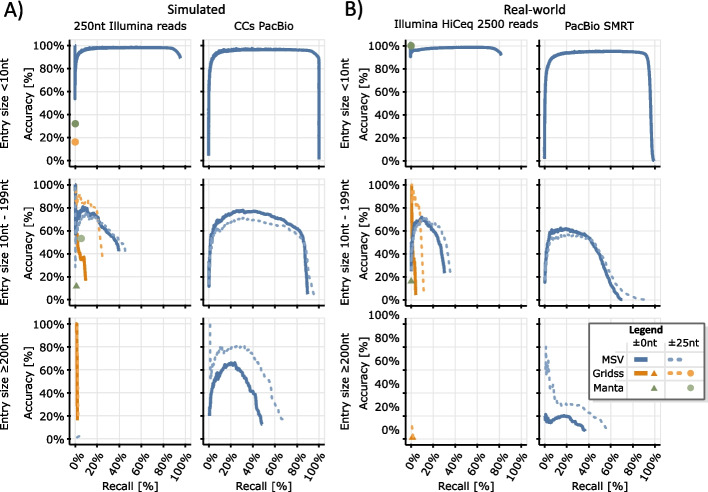
The figure shows the accuracy rate as a function of the recall rate for Gridss [30] (orange), Manta [3] (green), and our approach (blue) on the two yeast genomes YPS138 (as reference genome) and UFRJ50816 (as sequenced genome). Here the accuracy rate is the percentage number of correct entries among all reported entries. A reported entry is considered as correct if it matches the position of a ground truth entry. The recall rate is the number of correctly reported entries over the number of entries in the respective ground truth matrix. **A** shows benchmarking for simulated reads. There, we simulate 100 × coverage for 250-nt-long Illumina reads with DWGSIM [36] using default parameters, while setting the mutation rate to zero. Furthermore, we simulate 100 × coverage for CCS-PacBio reads using SURVIVOR [37] using default parameters. **B** displays benchmarking results for the original Illumina HiSeq 2500 and PacBio SMRT reads that were utilized for the assembly of UFRJ50816 in [23]. The solid curves ($\pm$ 0nt) and triangles benchmark the SV callers for their ability to rediscover the exact locations of breakends. The dotted curves ($\pm$ 25nt) and discs show the callers’ performance if we grant a tolerance of $\pm$ 25nt for the breakends positions. Here, if an SV caller reports multiple entries within the emerging 50nt window, we pick the entry with the highest score and discard all remaining entries. Manta does not report different confidences and hence appears as single points in the analysis. For all other callers, curves are inferred by gradually adding calls to the analyzed set, in reverse order of their confidence. Gridss and Manta are designed to work with short reads (Illumina reads) merely and are therefore excluded from the PacBio benchmarking. The reported VCF BND-tags of Manta and Gridss are translated into corresponding matrix entries for benchmarking

So far, for the generation of the ground-truth entries, the complete genome ${A}_{S}$ is used as one virtual error-free read. Now, we evaluate our approach using simulated reads, where we rely on SURVIVOR [37] and DWGSIM [36] for the generation of CCS-PacBio and Illumina reads, respectively. The error profiles for SURVIVOR’s CCS-PacBio read generation are sampled from Minimap2 alignments for the PacBio-MtSinai-NIST reads of HG002 (AJ Son) [38]. We use simulated reads instead of real-world reads to establish a proper ground truth. In the context of our benchmarking, the entries of the ground truth matrix ${M}_{T}$ are distributed over three submatrices: A matrix ${M}_{T,\ge 200}$ (comprising 337 entries) consisting of all entries in ${M}_{T}$ of size $\ge$ 200nt, a matrix ${M}_{T,10-199}$ (comprising 1329 entries), comprising all entries of size $[10{\text{nt}},200{\text{nt}})$ and a matrix ${M}_{T,<10}$ (comprising 43,804 entries) with all the remaining entries (size $<$ 10nt). Here the size of an entry is the maximum of the distance between an entry’s breakends on the reference genome and an entry’s weight, i.e., the length of an inserted sequence that might be part of the entry (e.g., for a basic insertion the distance of the breakends is 0 and the size of the entry is the length of the inserted sequence). Figure 5 shows the outcome of our benchmarking for all three matrices. Callers that report basic SV (e.g., Sniffles [1] and Delly [4] are excluded from the benchmarking due to the previously reported ambiguities affecting the representation of complex rearrangements via basic SV. Manta reports complex events via the BND-tag and nested events via basic SV. Hence, we could translate the basic SV-calls as well as the BND-calls into our matrix representation. The benchmarking indicates a superiority of our approach compared to its competitors. Particularly for PacBio reads, it performs well over the full range of all three matrices. Furthermore, we surpass the other callers with respect to the maximal recall rate except for ${M}_{T,\ge 200}$ with Illumina reads, where Gridss can recall 2% of the 337 entries. Altogether, Gridss and Manta show a blindness outside of the middle-sized genomic rearrangements defined by ${M}_{T,10-199}$. Aside from this blindness, they struggle to determine the exact locations of breakpoints (solid curves of Fig. 5) as indicated by their improved behavior for the more relaxed benchmarking, where a tolerance of $\pm$ 25nt is granted in the context of the breakend reporting. The slightly better accuracy of Gridss for recall rates of up to 25% with ${M}_{T,10-199}$ in the case of a $\pm$ 25nt tolerance indicates a weakness of our heuristics for filtering false positives. This mirrors the conclusion that assembly-based SV calling is excellent for filtering false-positives of [30]. Manta shows an improved behavior for Illumina reads of length 100 nt. Therefore, an additional benchmarking for 100nt Illumina reads is given in Additional file 6.

Next, we analyze real-world reads. To accomplish this, we rely on the PacBio SMRT and Illumina HiSeq 2500 reads that were originally employed to generate the UFRJ50816 assembly [23]. As a result, we no longer measure accuracy and recall rates in comparison to a ground truth, as the ground truth is now unknown. Instead, we evaluate the similarity to the output of the genome assembler used during the creation of the UFRJ50816 assembly. For Illumina HiSeq 2500 reads, our curves for both simulated and real-world reads show a high degree of similarity. For PacBio reads with an entry size < 10nt, we observe the same similarity. However, as the entry size increases the curves become more divergent. Larger entry sizes correspond to larger SV, which subsequently increases the probability of discrepancies between the SV caller and the assembler.

The outcome of SV calling should allow the reconstruction of the sequenced genome from the respective reference genome. Our genome mapping graph model (adjacency matrices) permits such a reconstruction as described in the methods section. We now evaluate the genomes that result from a reconstruction using matrix entries inferred from the sequenced genome as one long error-free read. With respect to this reconstruction, we compare our approach, in the following called MSV, with Gridss. Here we work with Illumina reads in combination with PacBio reads for MSV and Illumina reads by themselves for Gridss. As shown in the first row of Table 1, the calls from the sequenced genome deliver a perfect reconstruction of the sequenced genome, which justifies the use of these calls as ground-truth. With MSV, the identity between chromosomes of the reconstructed genome and the sequenced genome varies from 97.0 to 99.9%. Here, these identities are computed as $\frac{i}{\mathrm{min}(\left|Q\right|,\left|R\right|)}$, where $i$ represents the number of matches in a banded ( $\mathrm{bandwidth}=\;abs\left(\left|Q\right|-\left|R\right|\right)+10,000\;\mathrm{nt}$) Needleman Wunsch alignment with two-piece affine gap costs between the sequences $Q$ and $R$. As described in the methods section, the reconstruction requires a traversal for resolving ambiguities resulting from genomic repetitions. SV callers do not compute such a traversal. Therefore, as an approximation, we infer this traversal from the sequenced genome itself. In the following, we call this traversal the primordial traversal. This traversal is trivially computable by ordering the entries of the sequenced genome according to their position on the sequenced genome. However, the primordial traversal can contradict the entries of a caller. For MSV, such contradictions occur between 195 (0.5%) of the 38,113 entries used during the reconstruction. For Gridss, such contradictions occur for 73 out of 155 entries (47%). Here the total number of entries is much lower since the entries of Gridss are mainly in ${M}_{T,10-199}$. The primordial traversal could be replaced by a naïve traversal that always prioritizes non-implicit edges over implicit edges (see the first subsection of the methods section for the definition of implicit edges) and stops if multiple non-implicit edges originate from a visited vertex. For MSV, 94.5% of pairs of consecutive edges in the primordial traversal are part of such a naïve traversal as well, i.e., merely 5.5% of edge pairs of the primordial traversal are contributing. Comparing the naïve approach to the primordial traversal, 97.6% of pairs of consecutive edges are part of both traversals.

**Table 1:** The table reports the rates of identity between UFRJ50816 (sequenced genome) and its reconstructions via YPS138 (reference genome) in combination with the matrix entries computed by MSV and Gridds. For comparison purposes, in the last row, the identity between YPS138 and UFRJ50816 is reported for all chromosomes. Here, the identity between two chromosomes is the quotient of the number of matches between both chromosomes and the size of smaller chromosome. In each column, the entry showing the highest amount of identity is highlighted in bold, where the sequenced genome (first row) itself is excluded

	**Reconstruction with calls from**	**Chromosome**															
		**1**	**2**	**3**	**4**	**5**	**6**	**7**	**8**	**9**	**10**	**11**	**12**	**13**	**14**	**15**	**16**
**Identity to sequenced genome**	Sequenced genome	100%	100%	100%	100%	100%	100%	100%	100%	100%	100%	100%	100%	100%	100%	100%	100%
	MSV	**99.1%**	99.3%	**99.2%**	**99.9%**	**99.8%**	97.1%	**99.9%**	98.8%	**98.9%**	**98.6%**	**99.7%**	**99.9%**	**99.8%**	**99.7%**	**99.7%**	**99.9%**
	Gridss	96.5%	**99.5%**	95.8%	98.5%	88.7%	92.8%	99.2%	**99.4%**	98.3%	**98.6%**	99.0%	80.6%	64.0%	97.3%	99.5%	99.6%
	Reference genome	97.4%	85.8%	98.5%	72.5%	92.3%	**97.8%**	94.5%	97.7%	76.1%	97.6%	83.9%	90.6%	85.5%	88.3%	93.8%	91.6%

The use of weights is necessary during reconstruction since some differences between the reference genome and the sequenced genome (e.g., mutations and insertions) should not be expressed via sequences of the reference genome. In our case, sequences that are not covered by any seed are represented as weights. This implies that some duplications become weights due to the occurrence filtering (see methods section). Here, 90.6% and 93.8% of these weights are sequences of size one (mutations), while 0.6% and 0.4% of these weights are sequences of size $\ge 100$ nt (long insertions or long repetitive regions), for entries of the sequenced genome and MSV, respectively. For MSV, 4.5% of the reconstructed genome originates from weights (inserted sequences), where 37,683 of MSV’s entries comprise a weight. For the calls of the primordial traversal, 3.0% of the reconstructed genome originate from weights and 42,597 entries have weights. Large insertions connect to one of two special sentinel vertices as described in the methods section. However, in the context of our evaluation of Yeast, all insertions are fully enclosed by one PacBio read at least. Therefore, for all entries, the merging scheme described in Additional file 9 can be applied in the context of the entries’ scoring.

## Discussion

The yeast genomes used for our analyses are small compared to, e.g., the human genome. This raises the question of the applicability of our approach to larger, more complex genomes. Currently, the repetitiveness of complex genomes practically overloads the seed processing involved in our approach. For example, the human genome comprises many nontrivial sequences with more than 10,000 occurrences distributed over all its chromosomes. In our approach, all occurrences of such a sequence must be memorized by seeds for each sequenced read that comprises that sequence. The resulting amount of seeds for all reads can be staggering for complex large genomes and is best handled by extending our approach as described later. For the yeast genomes, this problem is still manageable. Benchmarking can be done on standard hardware without any extensions of our approach while still working with genomes showing complex, nested SV. Nevertheless, the repetitiveness of genomes generally poses a problem during the analysis of genomic data. Here, the repetitiveness of sequenced genomes affects our approach differently than the repetitiveness of reference genomes. A sequence that occurs once on the reference genome but many times on the sequenced genome equals one or several duplications. Such duplications create cycles in our graph model that can be resolved via a graph traversal. On the contrary, sections of a sequenced genome that match several locations on a reference genome are more challenging. Here it is necessary to select the correct location from many candidates on the reference. This selection could be done via alignments, where the alignment’s CIGARs provide the seeds used for the computation of matrix entries. In detail, each maximally extended stretch of consecutive matches within the CIGARs of the primary and all supplementary alignments can be translated into a single seed. Here, secondary alignments should be excluded for ensuring that seeds do not overlap on their read intervals. However, such an alignment-based seed generation would conceal many complex genomic rearrangements as shown in the results section. Therefore, it represents an unsatisfactory solution.

Instead of tackling the repetitiveness of a reference genome, via, e.g., an alignment-based seed generation as mentioned above, it would be advantageous to avoid such repetitiveness in the first place. This can be achieved by switching to graph genomes. Here a repetitive sequence can be represented by a single vertex (or a subgraph for nested repetitive sequences) and the repetitiveness is resolved by a traversal throughout the graph. Currently, the axes of our adjacency matrices correspond to sequential genomes and therefore all matrix entries that connect non-neighboring vertices (vertices that belong to non-neighboring nucleotides on a genome) represent SVs. However, our approach does not require such sequential genomes in the context of the adjacency matrix. Instead, we can use a graph genome as a reference by placing the vertices of the graph genome on the axes of the adjacency matrix. Compared to a sequential genome, the genomic rearrangements are now matrix entries that connect vertices not neighboring in the graph genome. Furthermore, the graph traversal that is computed for representing the sequenced genome can immediately be used as another path in the graph genome. Hence, our approach can be used for adding new specimens to graph-based pan-genomes. Such additions might imply a modification of the pan-genome graph’s structure if the sequenced genome indicates an additional level of repetitiveness. In this work, we use sequential reference genomes for two reasons: (1) Currently, we do not compute the traversal that delivers a sequenced genome. (2) A bootstrapping that provides repetition-free graph genomes as starting points is required.

**Fig. 6:**
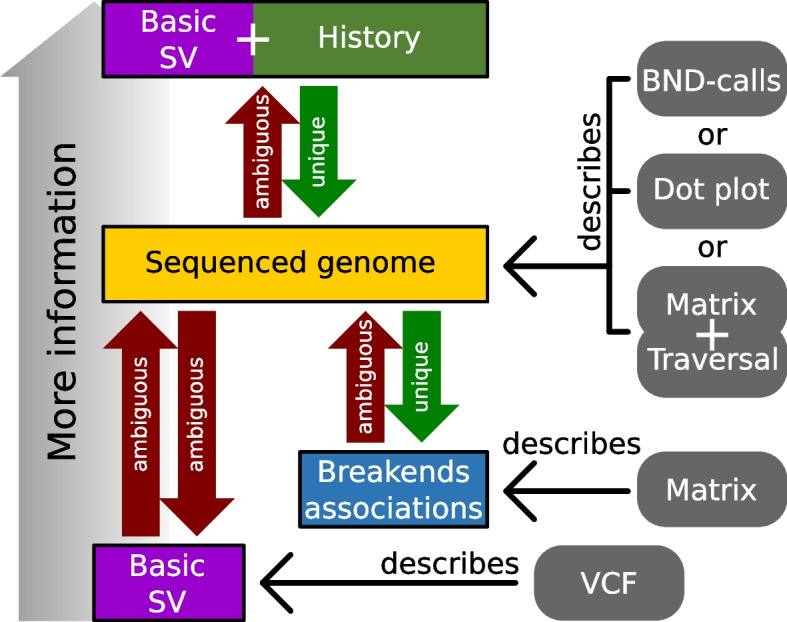
The figure visualizes several ambiguities inherent to the workflow of state-of-the-art SV calling. Furthermore, it places three SV description schemes (dot-plot, VCF-format, and adjacency matrix) into their context. Additional file 10 gives examples for the four shown ambiguities (red arrows) together with additional comments

In the results section, we investigate ambiguities resulting from the description of genomic rearrangements using basic SV (e.g., descriptions via the VCF format). Figure 6 extends this inspection of ambiguities to the relationships between genomic rearrangements, basic SV, sequenced genomes, and breakend associations: Evolutionary development is a stepwise process; its genetic state is usually observed via sequenced genomes, which are snapshots of a specimen’s genetic definition. Aside from some trivial cases, these snapshots cannot reveal the history of genomic alterations (basic SV) that leads from one snapshot to another because different histories can yield the same outcome. For example, three inversions can alter a sequence in the same way as one duplication followed by two deletions, e.g., shown in Additional file 10: Fig. S12 D). This inherent ambiguity, in turn, affects all basic SV-based description schemes, e.g., the VCF format. Therefore, any quantitative measurement of basic SV has to exclude nested variants (e.g., by using the benchmarking dataset proposed in [24]) or it is doomed to be ambiguous. For example, in [25–27, 39], these ambiguities are tackled by defining signature variant allele structures for several complex genomic rearrangements while ignoring all others. However, such complex genomic rearrangements are a significant aspect of the human genome as shown by Li et al. in [39], where they report that over half of all somatic variants in cancer genomes are nested and complex variants, and Collins et al. in [25], where they report that complex SV are abundant in non-cancer genomes as well. Furthermore, the repetitiveness of genomes in combination with the limited length of sequenced reads inflicts an additional level of ambiguity on the snapshots (sequenced genomes) themselves. For example, if a duplication’s size exceeds the length of all reads, the exact number of repeats can be guessed merely (see Additional file 10: Fig. S12 E). Our adjacency matrix-based approach resolves these ambiguities in two ways: (1) It abstracts from the history of genomic alterations. (2) It differentiates between the adjacency matrix itself and a traversal through its respective graph. In this context, the adjacency matrix captures the locations of all breakend pairs (see Additional file 1 and Fig. 6), while the traversal corresponds to the occurrence order of the breakend pairs on the sequenced genome. This traversal can only be computed if there are reads of sufficient length. However, for inferring adjacency matrix entries, short reads are sufficient since each entry merely requires two seeds to identify its breakend pair.

## Conclusions

We show that state-of-the-art approaches for SV calling are incapable of unambiguously representing nested and complex structural variants. This deficiency of current approaches leads to the exclusion of nested and complex variants in many contemporary studies (e.g., [25–27, 39]). This blind-spot in the analysis of genomic variation prohibits the association of complex and nested SV to diseases and observable traits.

For representing all forms of SVs unambiguously, we argue that one must express the outcome of genomic rearrangement instead of attempting to describe the process of rearrangement. Our outcome-focused approach leads to an adjacency matrix-based model that can cope with complex and arbitrarily nested SVs. This yields the following two advantages over current approaches for SV calling: (1) Detected genomic variations (from SNPs over isolated SVs to nested SVs) can be cross-compared between different individuals by inspecting overlapping matrix entries. (2) Our matrices can be easily translated into other forms of visualizations, e.g., dot plots. Furthermore, a practical evaluation of our approach using various yeast genomes demonstrates that our adjacency matrices can be efficiently computed from raw sequenced reads. Summarily, our work eliminates significant shortcomings of state-of-the-art SV calling and paves the way for the discovery and understanding of diseases and biological processes that are related to complex and nested SVs.

## Methods

### A skew-symmetric graph model for describing genomic rearrangements

**Fig. 7:**
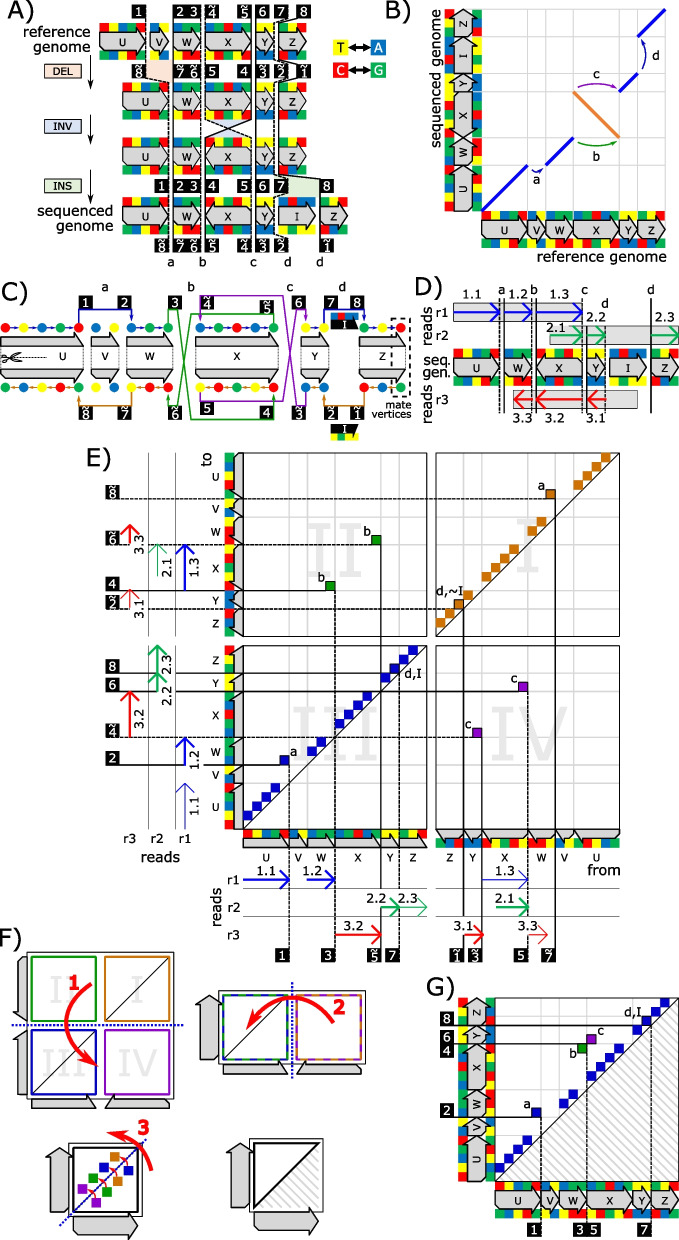
A visual overview of our approach for inferring a folded adjacency matrix from reads. **A** introduces a reference genome, a sequenced genome, and a history of basic SV (consisting of a deletion of the section $V$, inversion of $X$, and insertion of $I$) that transforms the former genome into the latter genome. The black-boxed numbers indicate the order of the breakends on the sequenced genome. A tilde over a number expresses that the corresponding breakend is on the reverse strand of the sequenced genome. The arrowheads of the genome sections $U$, $V$, $W$, $X$, $Y$, and $Z$ symbolize their direction on the reference; the colored boxes above and below are their nucleotides (see Additional file 2) on forward and reverse strand, respectively. **B** shows the genomic rearrangement of **A** in form of a diagrammatic dot-plot (details on these dot-plots are in Additional file 2). Each of the breakend pairs $a,b,c$, and $d$ of **A** is indicated via an equally labeled arrow. **C** displays the skew-symmetric graph for the genomic rearrangement of **B**. The dashed box on the graph highlights an exemplary pair of mate vertices. The labeled edges of the graph correspond to the equally labeled breakend pairs of **A**. The weights $I$ on the edges labeled $d$ represent the inserted sequence on the forward and reverse strands. **D** introduces three error-free reads $r1,r2$, and $r3$. Their locations on the sequenced genome are visualized via gray boxes and their MEMs are displayed by colored arrows. $I$ is not covered by seeds because it is an insertion. **E** comprises the unfolded adjacency matrix for the skew-symmetric graph in **C**. The matrix is inferred from the three reads of **D**, where the MEMs can be associated via their numbers. For example, the entry $a$ corresponds to the two breakends (1) and (2), which are discovered via the MEMs 1.1 and 1.2 of the read $r1$. The first and last seed of each read has no breakend on the *y*-axis and *x*-axis, respectively. Such seeds are distinguished by using thin arrows. The edge weights $I$ and $\sim I$ on the mate edges labeled $d$ denote the inserted sequences on the forward and reverse strands, respectively. The coloring scheme for the matrix entries memorizes the strand information of edges as described in the “Folding of Adjacency Matrices” chapter of the methods section. **F** visualizes the adjacency matrix folding scheme of our approach. A step-by-step description of the folding for the matrix in **E** is given in Additional file 11. **G** depicts the folded form of the matrix **E**. In the folded form, the forward and reverse strands are unified. Therefore, all equally labeled entries of the matrix in **E** appear as a single entry in **G**

We describe rearrangements between a reference genome $R$ and a sequenced genome $S$ using a weighted, directed skew-symmetric graph (skew-symmetric graphs are defined, e.g., in [40]). In our graph model, vertices represent nucleotides of $R$. Using the edges and their weights, we express $S$ in terms of $R$. Let $u$ and $v$ be two vertices. Furthermore, let $N\left(u\right)$ and $N\left(v\right)$ be their respective nucleotides: An edge from $u$ to $v$, indicates that $N\left(v\right)$ follows $N\left(u\right)$ on $S$. Hence, edges can either express equality between $S$ and $R$, by connecting the vertices of nucleotides that follow each other on $R$, or express differences (i.e., breakend pairs) by connecting the vertices of nucleotides that are nonconsecutive on $R$. For example, in Fig. 7C, the edge between the first two vertices of the segment $U$ on the forward strand indicates that the corresponding nucleotides appear consecutively on the sequenced genome. Accordingly, the edge labeled $a$ expresses that $W$ follows $U$ on the sequenced genome. For each position on the reference, we have one vertex on the forward strand and one vertex on the reverse strand. These vertices are called mates. Additionally, each edge $\left(u,v\right)$ has a mate edge that connects the mate vertices of $u$ and $v$ in opposite direction. In Fig. 7C, the forward-strand edge labeled $a$ has a mate on the reverse strand that connects the first vertex of $W$ with the last vertex of $U$. The concept of mates is part of the skew-symmetry of our graph model. Using strand-crossing edges, we can represent breakend pairs of inversions. For example, in Fig. 7C, the first breakend pair of the inversion of $X$ on the sequenced genome is represented by the green mate edges labeled $b$. Insertions are modeled using the weights of edges. Hence, the edge connecting $Y$ and $Z$ in Fig. 7C comprises $I$ as weight for modeling the insertion of the sequence $I$ on the forward strand. For representing a genome, it is necessary to have a set of traversals that accumulatively visit all non-isolated vertices of the graph at least once. Each traversal represents one chromosome of the sequenced genome.

The example reads of Fig. 7D and E demonstrate the inference of entries for an unfolded adjacency matrix in the idealized situation of error-free reads. For this purpose, we use Maximal Exact Matches (MEMs). MEMs are seeds that are maximally extended in either direction. Each entry is generated by the combination of the head (*x*-position of entry) and tail (*y*-position of entry) of two arrows, which correspond to the endpoints of two MEMs that occur consecutively on the respective read. For example, the MEMs labeled 1.1 and 1.2 in Fig. 7D and E create the entry labeled $a$ in quadrant $III$. If a read from the forward strand and a read from the reverse strand span over the same breakend pair, they create mate entries in the unfolded matrix. For example, the mate entries labeled $b$ result from read ${r}_{1}$ (on the forward strand) and read ${r}_{3}$ (on the reverse strand). The entries touching the diagonal on their bottom right corner can be directly inferred from the seeds, where such a matrix entry $(u,v)$ is formed by the pair of consecutive nucleotides $N(u),N(v)$ in the respective seed. We call these entries implicit because they are not related to SVs and can be neglected in practical implementations.

### Folding adjacency matrices

We now propose a folding scheme for unifying mate matrix entries (e.g., the entries labeled $b$ in Fig. 7E) by exploiting the skew-symmetry of our graph model. In the context of this folding, for each edge $(u,v)$, we store the strands of $u$ and $v$ via two annotations. Each annotation can receive the value $F$ or $R$ for the forward strand or reverse strand, respectively. In short, we write $FF,FR,RF$ or $RR$ for the strand annotations of both vertices of an edge. In the context of these annotations, we use the following coloring scheme: $FF$ = blue, $FR$ = green, $RF$ = purple, $RR$ = light brown. In the unfolded matrix, these annotations are intrinsically defined via the quadrant an edge appears in Quadrant $I\to RR$, Quadrant $II$ $\to FR$, Quadrant $III$ $\to FF$, Quadrant $IV$ $\to RF$ (see Fig. 6E). The annotations allow a reconstruction of the unfolded matrix out of the folded one.

The folding scheme is visualized in Fig. 7F and comprises the following three steps:

1. The top half of the matrix is mirrored to the bottom half of the matrix.
2. The right half of the resulting rectangular matrix is mirrored to the left half.
3. In the resulting matrix, all entries below the diagonal are mirrored to their corresponding mates above the diagonal. During this mirroring, the two binary annotations of entries are swapped and complemented ($FF\to RR,FR\to FR,RF\to RF,RR\to FF$). The weights of mirrored edges are reverse complemented. Entries on the diagonal that are annotated with $FF$ stay untouched, while all other entries on the diagonal are mirrored to themselves as described above.

In Fig. 7, the example matrix of subfigure E is folded into the matrix shown in subfigure G. The two breakpoints (two breakend pairs) of an inversion always correspond to two entries in the folded matrix, where one entry is annotated $FR$ and the other $RF$. For example, in subfigure G, the entries labeled $b$ and $c$ correspond to the inversion $X$. For isolated inversions, these two entries are always direct neighbors in the folded matrix and their distance to the diagonal indicates the size of the inversion. The deletion of $V$ is represented by the mate entries labeled $a$. In the unfolded matrix, one of these mates is annotated $FF$ and the other $RR$. The folding combines these two mates as shown in subfigure G. As with isolated inversions, the distance to the diagonal in the folded matrix indicates the size of isolated deletions. The insertion of $I$ is represented via the weights $I$ and $\widetilde{I}$(the reverse complement of $I$) on the mate edges labeled $d$. Once more, the matrix folding combines both mates.

### Computing a folded adjacency matrix from real-world reads

In the following, we infer an adjacency matrix from a given set of reads and a reference genome $R$. Initially, we compute the MEMs of all reads. Each pair of these MEMs that occur consecutively on a read creates a single entry in the adjacency matrix. Here multiple reads can create the same entry. For coping with this, we move to a multigraph model. Furthermore, the endpoints of MEMs can deviate slightly from actual breakends due to sequencing errors and the repetitiveness of genomes. Therefore, we include a concept of fuzziness by extending each matrix entry to an area around it. For finding true breakend pairs, we merge overlapping areas and later reduce them to single points. In the context of this merging, the size of these areas should be chosen so that we do not merge areas that belong to different breakend pairs. For dealing with large insertions that are not enclosed by any read, two sentinel vertices are added to our graph model.

#### Computing MEMs for a read via reseeding

The MEMs used for inferring the adjacency matrix are computed via a process that comprises three steps: (1) an initial computation of MEMs with respect to the whole reference $R$ and a given read, (2) a locally confined reseeding, and (3) the elimination of overlaps between MEMs on the same read.

For 1, we compute MEMs of a specific minimum size using Minimizers [41] and a merge-extend scheme [35]. In this context, we apply an occurrence filtering that eliminates hash table entries of Minimizers with too many matches on the reference genome or the sequenced genome. (This is required for handling the repetitiveness of the involved genomes. Please note that the sequenced genome is not directly available. Instead, we use all reads, which incorporate the sequenced genome.) In contrast to the commonly used occurrence filtering on the reference, the occurrence filtering on the sequenced genome is a novel addition. It is achieved in two steps: First, the number of occurrences is counted for all Minimizers on all reads. Next, we compute the reference positions for all minimizers with fewer occurrences than a given threshold, where this threshold is modulated by the average coverage of all reads.

For 2, matches smaller than the minimal size of Minimizers as well as matches for repetitive sections purged by the occurrence filtering are missing in the set of MEMs computed in 1. Many of these missing MEMs can be discovered via a locally confined reseeding that works as follows: First, we sort each read’s MEMs according to their start positions on the read. Afterward, we visit both endpoints of each MEM and search in a rectangular area that starts on the endpoint and extends away from the MEM on read and reference (see Fig. 4C). If a MEM’s successor or predecessor (on the read) extends into this rectangular area, we shrink the area so that it lies between the endpoints of both MEMs. The size of this area determines the minimal size of MEMs to be discovered by the reseeding using the following formula:

$$\left(1-025^\lambda\right)^{\left(w-\lambda\right)\ast\left(h-\lambda\right)}\geq99\%$$

Let $\lambda$ denote the minimal size of MEMs. $w$ and $h$ are the width and height of the rectangular area for reseeding, respectively. $\lambda$ is chosen so that the probability of a random match of size $\lambda$ in an area of size $w*h$ is lower than 1%. For each rectangular area, we reseed by computing a single-use Minimizer index and by utilizing the merge-extend scheme proposed in [35] for turning the Minimizers into MEMs. The above procedure is repeatedly applied until it does not deliver new MEMs.

For 3, both of the above steps can deliver MEMs that overlap on a read. (These MEMs must originate from the same read.) However, as shown in the results section, such overlapping MEMs lead to erroneous matrix entries. We solve this problem by applying a seed shortening, where we distinguish between two kinds of situations as follows: A) A MEM ${m}_{1}$ is fully enclosed by another MEM ${m}_{2}$ on the read. In this case ${m}_{1}$ is deleted and ${m}_{2}$ is kept unaltered. B) The endpoint of ${m}_{1}$ is above the start point of ${m}_{2}$, but ${m}_{1}$ is not fully enclosed by ${m}_{2}$. So, we determine the central point ${p}_{cut}$ of the overlap between both seeds and shorten both seeds with respect to ${p}_{cut}$. (The endpoint of ${m}_{1}$ is moved to ${p}_{cut}-1$; the startpoint of ${m}_{2}$ is moved to ${p}_{cut}$.) The described overlap elimination is applied on SoCs as well. Additional file 12 visualizes both forms of overlap elimination and extends the explanation.

#### Fuzzy inference of edges from MEMs

Our fuzziness concept exploits the spatial locality of neighboring vertices on the reference genome. (Neighboring vertices (and so adjacency matrix entries) correspond to neighboring nucleotides on the reference.) In the following, we represent a MEM as a triple $(q,r,l)$, where $q$ is the MEM’s start on the read (query), $r$ is the MEM’s start on the reference and $l$ is the length of the MEM. Let ${m}_{1}=({q}_{1},{r}_{1},{l}_{1})$ and ${m}_{2}=({q}_{2},{r}_{2},{l}_{2})$ be two MEMs that occur consecutively on some read. These two MEMs create a matrix entry $e=\left({v}_{x},{v}_{y}\right)$ with ${x=r}_{1}+{l}_{1}$ and $y={r}_{2}$. Here, the vertex ${v}_{i}$ corresponds to the $i$ th nucleotide of the reference genome. Furthermore, we assume the existence of a corresponding true entry, denoted by $T(e)$, which emerges from error-free reads. The entry $e$ can deviate from $T(e)$ due to sequencing errors that shorten or extend MEMs erroneously. Here a shortened MEM causes a deviance leftwards (${m}_{1}$ shortened) or upwards (${m}_{2}$ shortened) and an enlarged MEM causes deviance rightwards (${m}_{1}$ extended) or downwards (${m}_{2}$ extended). An erroneous extension of $n$ nucleotides requires a wrongful match between reference and read of $n$ nucleotides, which is caused by $n$ consecutive sequencer errors. On the contrary, a shorting by $n$ nucleotides can be caused by a single sequencer error that breaks a MEM into two pieces, where the small piece (required for the entry) gets lost due to size constraints or occurrence filtering. This implies that an erroneous shortening is more probable than an erroneous enlargement. For dealing with such deviations of entries, we define a rectangular area around each entry $e=({v}_{x},{v}_{y})$ that is expected to include $T(e)$ as follows: For denoting a rectangular matrix area $A$, we use a quadruple $(a,b,w,h)$, where $a,b$ are the coordinates of the top-left point of $A$ and $a+w,b-h$ are the coordinates of the bottom-right point of $A$. We define a rectangular area $A\left(e\right)=(x-\sigma ,y-\sigma ,f+\sigma ,f+\sigma )$, where $f$ and $\sigma$ deal with the erroneous shortening or enlargement of MEMs, respectively. $A(e)$ is called the entry area of $e$ and we choose $f=\mathrm{min}(\left|x-y\right|,10)$ as well as $\sigma =3$ by default. Additional file 13 visualizes the concept of entry areas. All entry areas $A\left(e\right)$ inherit a strand annotation from their respective entry $e$. The folding of the adjacency matrix automatically mirrors entry areas and adapts their strand annotations as required. Accordingly, in the folded matrix, an entry area can extend into four different directions with respect to its origin. For coping with the high density of entries immediately above the diagonal of the adjacency matrix, the fuzziness parameter $f$ is downregulated in this region (see Additional file 14). For an adjacency matrix $M$, entry areas in $M$ correspond to bipartite subgraphs of $M$’s graph.

#### Computing clusters by merging overlapping entry-areas

Let $M$ be a matrix and $E$ be the maximal set, where we have $T\left(e\right)=T({e}{\prime})$ for all pairs $e,{e}{\prime}\in E$. This entry, which is equal for all elements in $E$, is called the true entry of $E$ and denotes it by $T(E)$. If the parameters $f$ and $\sigma$ are chosen properly, the entry area $A(e)$ overlaps $T(E)$ for all entries $e\in E$. This implies that all these entry areas mutually overlap. Hence, we can compute the set $E$ (without knowledge about the true entry $T(E)$ itself) by joining overlapping entry areas of $M$ into clusters. Such clustering can be performed efficiently by a single line-sweep as described in Additional file 14. Additional file 13: Fig. S15 B) shows an example for the clustering of several entry areas that belong to the same true entry. As described above, the matrix folding maps areas originating from the reverse strand into the corresponding areas of the forward strand. The clustering is performed after the folding so that entry-areas from forward and reverse strand reads are unified. The weights (inserted sequences) of matrix entries in $E$ can be different. In this case, multiple sequence alignments can be used for obtaining a unified weight. So far, we skip such multiple sequence alignments and leave the weight empty instead.

#### Approximating true entry locations

We now use all entries in $E$ for computing an approximation of $E$’s true entry $T(E)$. For this purpose, we compute two sets $X$ and $Y$ as follows:$X=\left\{i \right| ({v}_{i},\_)\in E\}$,$Y=\{ j |(\_,{v}_{j})\in E \}$. Depending on the two strand-annotations of $E$ (these must be equal for all $e\in E$), the approximation of $T\left(E\right)=\left({u}_{x},{u}_{y}\right)$ is defined as follows:

- $FF$: $x$ is the ${95}^{\mathrm{th}}$ percentile of $X$ and $y$ is the ${5}^{\mathrm{th}}$ percentile of $Y$
- $FR$: $x$ is the ${95}^{\mathrm{th}}$ percentile of $X$ and $y$ is the ${95}^{\mathrm{th}}$ percentile of $Y$
- $RF$: $x$ is the ${5}^{\mathrm{th}}$ percentile of $X$ and $y$ is the ${5}^{\mathrm{th}}$ percentile of $Y$
- $RR$: $x$ is the ${5}^{\mathrm{th}}$ percentile of $X$ and $y$ is the ${95}^{\mathrm{th}}$ percentile of $Y$

Here, the first annotation determines whether $x$ is the ${95}^{\mathrm{th}}$ (for $F$) or ${5}^{\mathrm{th}}$ (for $R$) percentile of $X$, while the second annotation sets $y$’s percentiles reciprocally ($F:{5}^{\mathrm{th}}$, ${R:95}^{\mathrm{th}}$) regarding $Y$. The strand annotation-dependent percentile scheme is required due to the abovementioned mirroring of entry areas (in the context of the matrix folding scheme). Due to this mirroring, an entry can expand into an area in four different directions.

#### Large insertions and the sentinel vertex

Let $I$ be a large insertion and let $r$ be a read that covers $I$ partially at the beginning or end without fully enclosing $I$. In this case, an edge corresponding to $I$ cannot be formed from $r$ because either the edge’s origin vertex or destination vertex is unknown. Accordingly, it is impossible to create a valid adjacency matrix entry for $I$ using $r$. For coping with this situation, a sentinel vertex and its mate (one for the forward strand and one for the reverse strand) are added to our graph model. These sentinels take the position of the missing origin or destination vertex in the context of the edge creation. As for all vertices, one row and one column of the folded adjacency matrix correspond to the sentinel vertex and its mate (see Additional file 9).

Practically, edges are connected to the sentinel vertex or its mate if the outer ends of a read exceed a given distance to the outmost ends of its seeds (MEMs). Furthermore, the previously introduced clustering for adjacency matrix entries can be used with the sentinels’ column and row as well. If there are reads that fully enclose $I$, a merging with entries that only partially enclose $I$ is possible. If such fully enclosing reads are absent, genome assembly techniques are required for the reconstruction of $I$ and its matrix entry. Additional file 9 comprises a detailed description of the creation, clustering and merging of edges that connect to the sentinel vertex or its mate.

#### Computing confidence scores for matrix entries

We compute the confidence score $Conf(E)$ of an adjacency matrix entry $E$ at the position $({u}_{X}, {u}_{Y})$ as $Conf\left(E\right)=Reads(E)/(Cov\left(X\right)+Cov\left(Y\right))$. Here, $Reads(E)$ is the number of reads that contribute at least one entry-area to the cluster belonging to $E$, where entry-areas are the fuzzy edges inferred directly from MEMs. We define coverage $Cov\left(Z\right)$ as the number of reads that have at least one seed overlapping the $Z$ th nucleotide of the reference. The subterm $Cov\left(X\right)+Cov\left(Y\right)$ of the above computational scheme represents the sum of coverages from the $X$ and $Y$ matrix position of $E$.

### Reconstructing sequenced genomes from graphs

Highly accurate SV calling should allow a reconstruction of the sequenced genome on the foundation of the reference genome and the SV calls. In the following, we assume the existence of a sequenced genome $S$ together with a set $X$ of simulated long (PacBio) and short (Illumina) reads for $S$. Using our proposed approach, we compute an adjacency matrix ${M}_{S}$ using $S$ as one single error-free read and a matrix ${M}_{X}$ using the set of reads $X$. The matrices ${M}_{S}$ and ${M}_{X}$ can differ slightly due to the impact of sequencing errors, the size of reads in $X$, and the repetitiveness of the reference genome. The differences between ${M}_{S}$ and ${M}_{X}$ can manifest in three ways: (1) an entry of ${M}_{S}$ is slightly misplaced in ${M}_{X}$, (2) An entry of ${M}_{S}$ is missing in${M}_{X}$, and (3) ${M}_{X}$ comprises an additional entry that does not occur in ${M}_{S}$. The matrices ${M}_{S}$ and ${M}_{X}$ define two skew-symmetric genome-mapping graphs ${G}_{S}$ and ${G}_{X}$, respectively. Let ${t}_{S}$ be a traversal through ${G}_{S}$ that visits the edges in compliance with $S$. ${t}_{S}$ defines a set of tuples ${T}_{S}=\{ \left(i,e\right):$ $e$ is the $i$ th edge visited during the traversal ${t}_{S} \}$. Here an edge can be part of multiple tuples in ${T}_{S}$. By following the edges in ${T}_{S}$ in ascending order of their $i$-values, we can reconstruct $S$ in terms of ${G}_{S}$. In this context, the diagonal entries (which are not part of the matrix in practice for efficiency reasons) are implicitly inserted. According to the computation of ${t}_{S}$ on the foundation of $S$, it must be possible to compute a traversal ${t}_{X}$ through ${G}_{X}$. For this purpose, the partial traversal information comprised in reads needs to be accumulatively combined using techniques known from genome assemblers. Furthermore, insertions that are not fully covered by any read require read-to-read alignments for reconstruction. These two problems are subject to further research. In this work, we instead infer ${T}_{X}$ directly from ${T}_{S}$ as follows: For each pair $\left(i,e\right)\in {T}_{S}$, we search for the spatially closest neighbor $e{\prime}\in {M}_{X}$. If the distance to $e{\prime}$ exceeds a given threshold, we ignore the pair $(i,e)$. Otherwise, we add a tuple $(i,e{\prime})$ to an initially empty ${T}_{X}$. In the context of this addition, we copy the weight (weights are insertions) of $e$ to $e$’ too. Additionally, we define a successor function on the indices in ${T}_{X}$ as$succ\left(i\right)=\mathrm{min}\left(\left\{j \right| j>i {\text{and}} \left(j,e\right)\in {T}_{X}\right\})$. For example, in Additional file 8, we have $succ\left(1\right)=2$ and $succ\left(2\right)=4$ for ${T}_{X}$. The reconstruction of an approximated $S$ via ${T}_{X}$ follows the same algorithmic approach as described for $S$ and ${T}_{S}$. However, due to the differences between ${M}_{S}$ and ${M}_{X}$, we get an approximated form of $S$ merely. As shown in Additional file 8, these differences can cause a situation, where two tuples $\left(i,e\right)$ and $\left(succ\left(i\right),e{\prime}{\prime}\right)$ in ${T}_{X}$ contradict the graph ${G}_{X}$. (I.e., the origin of the edge $e{\prime}{\prime}$ cannot be reached from the destination of the edge$e$.) In this case, we continue the reconstruction at the origin of $e{\prime}{\prime}$ as soon as we reach the destination of$e$. For example, in Additional file 8, this contradiction occurs between the edges $a{\prime}$ and $b{\prime}$ as well as $b{\prime}$ and $d{\prime}$. Therefore, the reconstruction comprises the segments before the destination of $a{\prime}$ and the segments following the origin of $d{\prime}$ merely. By choosing the origin of $d{\prime}$ instead of its destination, we avoid a bypassing of the insertion $I$.

As mentioned before, our skew-symmetric graph model unifies forward and reverse strand. The reconstruction process can encounter edges connecting vertices of opposite strands that we call crossing edges. Due to such crossing edges, the strand must be tracked during reconstruction. In ${T}_{S}$ this tracking is achieved by inverting a “current strand”-variable whenever the reconstruction passes crossing edges. (The current strand must be considered during the abovementioned insertion of implicit edges; see Figs. 6E and 7C.) However, in ${T}_{X}$, the strand tracking can fail due to the disappearance of crossing edges. For tackling this shortcoming, we inspect ${T}_{S}$ whenever we resolve one of the abovementioned contradictions between ${T}_{X}$ and ${G}_{X}$.

## Supplementary Information

**Additional file 1.** Breakends versus Breakpoints.

**Additional file 2.** Diagrammatic dot-plots and genome section pictograms. Contains Fig. S1.

**Additional file 3.** Unfolded matrices and full graphs for Fig. 1 of themain text. Contains Fig. S2 and S3.

**Additional file 4.** Experimental setup for the generation of nested genomic rearrangements. Contains Fig. S4.

**Additional file 5.** Description of the SV caller prototype and benchmarking environment.

**Additional file 6.** SVs that are hidden to aligners – Extended analysis. Contains Fig. S5, S6, and S7.

**Additional file 7.** Computation of ground truth entries and evaluation of 100nt long Illumina as well as Oxford Nanopore reads. Contains Fig. S8 and S9.

**Additional file 8.** Reconstructing sequenced genomes from graphs. Contains Fig. S10.

**Additional file 9.** The sentinel vertex. Contains Fig. S11 and Table S1.

**Additional file 10.** Ambiguities inherent to basic SV. Contains Fig. S12.

**Additional file 11.** Detailed matrix folding of Fig. 7 in the methodssection. Contains Fig. S13.

**Additional file 12.** Detailed description of overlap-elimination. Contains Fig. S14.

**Additional file 13.** Fuzzy inference of edges from MEMs. Contains Fig. S15.

**Additional file 14.** Line-sweep based clustering of entry-areas. Contains Fig. S16 and S17.

**Additional file 15.** Review history.

## Data Availability

An open-source, MIT-licensed prototype implementation of our approach can be found at https://github.com/ITBE-Lab/MA [42, 43]. The scripts used for performing all experiments are available at https://github.com/ITBE-Lab/MSV-EVAL [44, 45]. The PacBio reads and Yeast genomes used and analyzed in the current study have been created by [23] and are available from the European Nucleotide Archive and Genebank under the accession code PRJEB7245 [46, 47]. The Illumina reads used and analyzed in the current study have been created by [23] and are available from the Short Reads Archive under the accession code PRJNA340312 [48].
