# Supplementary material for: MSV: a modular structural variant caller that reveals nested and complex rearrangements by unifying breakends inferred directly from reads
Source: Genome Biol. 2023 Jul 17;24:170. doi: 10.1186/s13059-023-03009-5 (PMC10351204; doi:10.1186/s13059-023-03009-5)
Supplement: Supplementary file 1 — Additional file 1. Breakends versus Breakpoints. [file 13059_2023_3009_MOESM1_ESM.docx]

# Additional file 1: Breakends versus Breakpoints

Often the notion ‘breakpoint’ is used in the context of SV calling for identifying positions on the reference genome and reads [6, 7]. The number of breakpoints varies for different types of SV. For example, an insertion has two breakpoints on the read and one breakpoint on the reference, while a deletion has one breakpoint on the read and two breakpoints on the reference. In the context of our proposed approach, we aim for a description of SV that does not use basic SVs. Therefore, we need terminology that expresses the endpoints of SV independent of their type. For this purpose, we rely on the notion ‘breakend’ as introduced in the “Specifying complex rearrangements with breakends” section of the “The Variant Call Format (VCF) Version 4.2 Specification”. In contrast to breakpoints, two breakends can occur in the same position on reference or read. Accordingly, all basic SVs are characterized by exactly four breakends (two on the read and two on the reference). Here we use the notion breakend pair for referring to the respective two breakends on read or reference.
