# Supplementary material for: MSV: a modular structural variant caller that reveals nested and complex rearrangements by unifying breakends inferred directly from reads
Source: Genome Biol. 2023 Jul 17;24:170. doi: 10.1186/s13059-023-03009-5 (PMC10351204; doi:10.1186/s13059-023-03009-5)
Supplement: Supplementary file 2 — Additional file 2. Diagrammatic dot-plots and genome section pictograms. Contains Fig. S1. [file 13059_2023_3009_MOESM2_ESM.docx]

# Additional file 2: Diagrammatic dot-plots and genome section pictograms

**Figure S1.** Diagrammatic dot-plots and genome section pictograms.

Subfigure **A)** shows a dot-plot, where each blue box indicates a match on identical strands and each orange box indicates a match on opposite strands. The reference genome and the sequenced genome appear on the x-axis and y-axis, respectively. For the reference genome, the colored squares on top of the sections $U,V$ represent the nucleotides on the forward strand. Accordingly, the reverse strand appears below the sections. The forward strand of the sequenced genome is on the left of its genome section, while the reverse strand is on its right.

Subfigure **B)** displays a diagrammatic representation of the left side’s dot-plot. In this diagrammatic representation, consecutive matches (on equal as well as opposite strands) appear as lines, where the start of the line includes the bottommost match and the end includes the topmost match. The yellow, red, blue and green squares on the reference genome and sequenced genome represent the nucleotides T, C, A and G, respectively.

Subfigure **C)** explains our visualization for genome sections using arrow-like pictograms. A pictogram can comprise a nook, which indicates that its nucleotide sequence occurs on the reference. This nook in combination with the arrowheads expresses the direction of the pictogram’s nucleotide sequence on the reference genome. Via this scheme, we visualize that genomic inversions are $180^{\circ}$ rotations (and not a mirroring operation).
