# Supplementary material for: MSV: a modular structural variant caller that reveals nested and complex rearrangements by unifying breakends inferred directly from reads
Source: Genome Biol. 2023 Jul 17;24:170. doi: 10.1186/s13059-023-03009-5 (PMC10351204; doi:10.1186/s13059-023-03009-5)
Supplement: Supplementary file 3 — Additional file 3. Unfolded matrices and full graphs for Fig. 1 of themain text. Contains Fig. S2 and S3. [file 13059_2023_3009_MOESM3_ESM.docx]

# Additional file 3: Unfolded matrices and full graphs for Fig. 1 of the main text

**Figure S2.** The figure shows that a duplication followed by two inversions can lead to the same genomic outcome as two duplications followed by one inversion. The respective unfolded adjacency matrix and the full graph are displayed in the figure below.

**Figure S3.** Fig. 1 of the main text depicts several ambiguities inherent to the description of genomic rearrangements using basic SV. The main text’s figure merely shows simplified instances of our graph model and their folded adjacency matrices.

The above figure displays the unfolded matrices together with their respective full skew-symmetric graphs. Subfigure A) and B) correspond to Fig. 1 A) and B) of the main text, respectively. Subfigure C) corresponds to Figure S2. The folding scheme for matrices is described in the methods section of the main text. Black outlined entries correspond to the equally colored edges of their respective graph. All other entries belong to dashed edges (within the genome sections $A,B,C,D$) and are not shown in Fig. 1 of the main text.

Due to the folding scheme for matrices, the outlined blue and orange matrix entries in C) correspond to the single unlabeled blue entry in the respective folded matrix. There, the “from-to” direction of this unlabeled blue entry does not match the direction of the corresponding edge in the simplified graph. The same apparent paradox affects the edge labeled $b$ in Fig. 1 B). The unfolding of the matrix always resolves such paradoxes (see the above subfigure B and C).
