# Supplementary material for: MSV: a modular structural variant caller that reveals nested and complex rearrangements by unifying breakends inferred directly from reads
Source: Genome Biol. 2023 Jul 17;24:170. doi: 10.1186/s13059-023-03009-5 (PMC10351204; doi:10.1186/s13059-023-03009-5)
Supplement: Supplementary file 4 — Additional file 4. Experimental setup for the generation of nested genomic rearrangements. Contains Fig. S4. [file 13059_2023_3009_MOESM4_ESM.docx]

# Additional file 4: Experimental setup for the generation of nested genomic rearrangements

**Figure S4.** Visualization of the generation of nested genomic rearrangements.

Ambiguities that are inherent to the description of genomic rearrangements via basic SV are evaluated by applying the following four steps:

1. A specific (generated) genomic rearrangement is represented using a diagrammatic dot-plot.
2. The resulting diagrammatic dot-plot is turned into error-free alignments (incl. CIGAR).
3. The error-free alignments are forwarded to various SV callers (Sniffles etc.) for SV detection.
4. The detected SVs of the respective SV callers are evaluated and visualized.

These steps are explained in detail now:

1) We use the human genome (GRCh38.p12) as the reference genome; the length parameter $l$ (see above figure) is set to $1000$ nt. The length of the sections labeled $A,B,C$ of Fig. 1 of the main text are equal to the sizes of the respective sections in the above figure (although differently visualized in Fig. 1). The increased size of the sections $A$ and $C$ compared to $B$ creates SV-free regions for supporting the sampling step that is part of some SV callers (e.g. Delly). Each diagrammatic dot-plot is manually translated into a set of pseudo-seeds $X$ that incorporates the dot-plot’s genomic rearrangement, where a pseudo-seed is a quadruple $(q,r,l,\xi)$ of a query position $q$, reference position $r$, length $l$ and a value $\xi\in\{same,opposite\}$ that indicates equivalence between same or opposite strands. (Same strand and opposite strand pseudo-seeds are visualized as blue lines and orange lines, respectively.) All manually created pseudo-seeds are maximally extended in either direction. After creation, all pseudo-seeds in $X$ are moved by a random distance $d$ on the reference genome’s first chromosome. For the above example, this delivers the set $Y=\{\left( 0,d,11l,same \right),\left( 11l,10l+d,l,opposite \right), \left( 12l,11l+d,10l,same \right)\}$. The resulting set $Y$ defines the simulated sequenced genome. Insertions are injected by creating random sequences (Mersenne Twister random generator) of appropriate size. In the above example, the simulated sequenced genome is $AB\tilde{B}C$ for the reference genome $ABC$.

2) For generating alignments, intervals are picked on the simulated sequenced genome from 1). For long-read alignments, these intervals are of size $2*l-1$ (so that alignments cover no more than two breakend-pairs). For paired short read alignments, we rely on interval pairs, where each interval has a size of 250 nt and the distance between a pair is 100 nt. Using a sequenced genome interval $I$, an alignment is computed as follows:

We create a copy $Y'$ of $Y$ and limit the pseudo-seeds in $Y’$ to the interval $I$ afterward. For this purpose, we delete all pseudo-seeds in $Y'$that are completely outside of $I$ and shorten seeds that cross $I$’s endpoints. The remaining pseudo-seeds in $Y'$ are used for the creation of alignments. Here each seed results in a single alignment with a CIGAR comprising a match equally sized to the length of the pseudo-seed. Among these, all alignments except for the longest one are marked as supplementary. The reference position of the alignments is given by their pseudo-seed’s reference positions. The query (read) position of the alignments is the relative distance of their pseudo-seeds to the beginning of $I$. The simulated read is the sequence in $I$ on the simulated sequenced genome. The strand of an alignment is given by the strand of its pseudo-seed. The above scheme for alignments and reads generation is used for generating a coverage of 100 (within the reference genome’s section $ABC$).

3) All alignments generated in step 2) are collected in a SAM file that is forwarded to the respective SV caller.

4) By reading the fields ‘ALT’, ‘POS’ and ‘INFO->END’, the VCF-output of the SV caller is visualized as labeled intervals on the reference genome. Here the order in the diagrams is equal to the order of their respective occurrence in the VCF-file.

Our prototype (see Additional File 6) computes all folded matrices exactly as shown in Fig. 1 of the main text.
