# Supplementary material for: MSV: a modular structural variant caller that reveals nested and complex rearrangements by unifying breakends inferred directly from reads
Source: Genome Biol. 2023 Jul 17;24:170. doi: 10.1186/s13059-023-03009-5 (PMC10351204; doi:10.1186/s13059-023-03009-5)
Supplement: Supplementary file 5 — Additional file 5. Description of the SV caller prototype and benchmarking environment. [file 13059_2023_3009_MOESM5_ESM.docx]

# Additional file 5: Description of the SV caller prototype and benchmarking environment

All evaluation and analysis of our approach is performed by using a prototype implementation that is characterized as follows:

- Similar to the approach of the Modular Aligner MA [1] and TensorFlow, Python 3 is used for constructing a computational graph that redirects all computational operations to a C++ library (realized in C++-17). This architecture keeps Python inactive during graph execution for gaining high performance and exploiting parallel computing (multithreading) advantages.
- For gaining a high level of flexibility, the C++ library, in turn, relies on a PostgreSQL database as a backend. The database is used for storage purposes (e.g. reads, adjacency matrix) and efficient data retrieval operations using SQL.
- For visualizing purposes, an additional viewer application is realized using the Python-Bokeh library. On the database level, the viewer exploits the highly efficient storage and retrieval of spatial data offered by PostGIS.
- The prototype is publically available on GitHub under the MIT license on

https://github.com/ITBE-Lab/MA

- Our evaluation framework is publically available on GitHub under the MIT license on

https://github.com/ITBE-Lab/MSV-EVAL

All experimental work is done on an *AMD Ryzen Threadripper 1950X 16-Core Processor* with 128 GB RAM.

The versions of third-party software and applications are listed below:

- Minimap2 2.25-r1173
- Delly v0.8.1
- Sniffles 1.0.8
- NGMLR 0.2.8
- Postgres 12.3
- Postgis 3
- MA 2.0.2-a09a1f5
- Gridss 2.12.2
- Manta 1.6.0
- GraphAligner 1.0.15 (We were unable to analyze versions 1.0.17b, 1.0.17, and 1.0.16 due to their attempts to allocate more RAM than our system's available capacity (126 GB).)
