# Supplementary material for: MSV: a modular structural variant caller that reveals nested and complex rearrangements by unifying breakends inferred directly from reads
Source: Genome Biol. 2023 Jul 17;24:170. doi: 10.1186/s13059-023-03009-5 (PMC10351204; doi:10.1186/s13059-023-03009-5)
Supplement: Supplementary file 6 — Additional file 6. SVs that are hidden to aligners – Extended analysis. Contains Fig. S5, S6, and S7. [file 13059_2023_3009_MOESM6_ESM.docx]

# Additional file 6: SVs that are hidden to aligners – Extended analysis

## Simulating sequencing errors

To obtain error rates and indel sizes consistent with state-of-the-art sequencers, we sample the alignments from the publicly available bam files at <https://downloads.pacbcloud.com/public/revio/2022Q4/HG002-rep1/analysis/>. These bam files contain alignments of PacBio HiFi revio system reads sequenced from the HG002 cell line. The reads are aligned to GRCh38.p12 using the pbmm2 aligner available at https://github.com/PacificBiosciences/pbmm2. Using this context, following the description in [22], we extract substitution, insertion, and deletion probabilities, as well as insertion and deletion sizes, from the alignments' CIGAR strings.

We begin the creation of a simulated read by selecting a random section of the genome GRCh38.p12 as the initial sequence. Next, we induce a specific structural variant to this section. Finally, we incorporate simulated sequencing errors to generate the simulated read. When inducing sequencer errors, insertions and deletions from the sequencer can alter the query positions of breakends from the structural variant. Insertions push the breakend positions forward on the read, while deletions push them backward. Therefore, since we only consider a breakend as rediscovered when the aligner identifies its reference and query positions, we need to adjust the query positions of breakends according to the simulated indels. (Technically, we store the position and size of an indel to perform these adjustments.) It is important to note that the reference positions of breakends remain unaltered throughout the process. Additionally, breakends that are covered by a simulated (sequencer-error-) deletion are removed from the list of breakends requiring rediscovery. (This removal is justified by the fact that simulated sequencer error deletions can be quite large. If a large simulated deletion removes the query region around a breakend, that breakend effectively becomes invisible to the aligner.)

## Analyzing micro-translocations


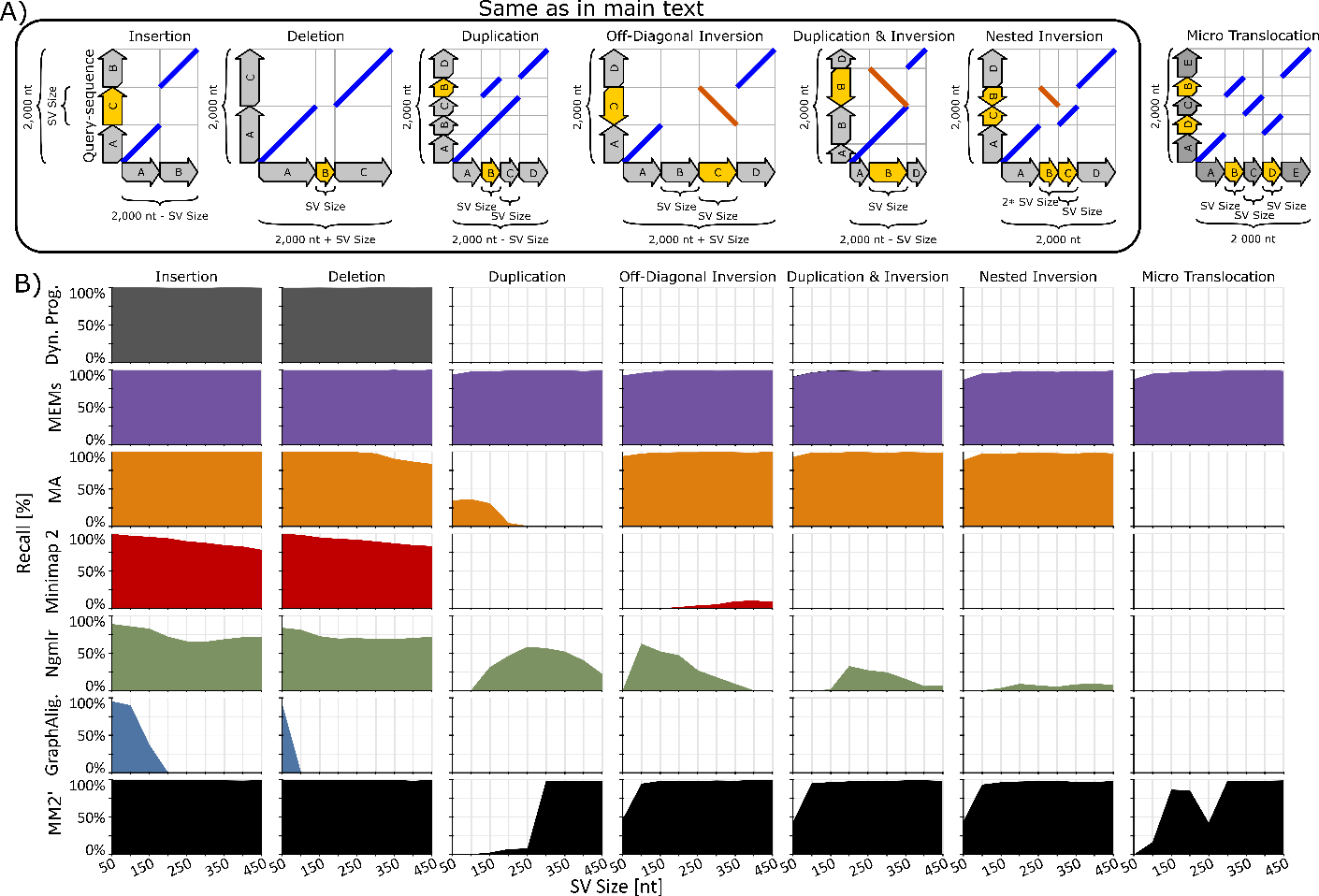


**Figure S5.** In addition to Fig. 2 of the main text, this figure analyses micro translocations on sequencer error-free queries and comprises curves that display the recall rates for Minimap2 with a special setup that avoids discarding chains (MM2’). The detailed parameters for MM2’s setup are: “-z 400,1 --splice -P”. The setting of the “-z” parameter to “400,1” decreases the z-drop, which, in turn, improves the discovery of inversions at the cost of increased runtime and more false positives. The “--splice” parameter enables splice mode. The “-P” instructs Minimap2 to keep all chains for the price of tremendously increased runtimes and excessively large SAM files.

For micro translocations, all aligners fail except Minimap2 with the above special setup. This behavior can be explained by the path-contradicting nature of this kind of genomic rearrangement. The different behavior of Minimap2 for the two settings (red curve and black curve) indicates that, for getting reasonable runtime, Minimap2’s heuristics (z-drop, chaining, etc.) remove information vital to the discovery of genomic rearrangements.

## Analyzing additional parameter settings


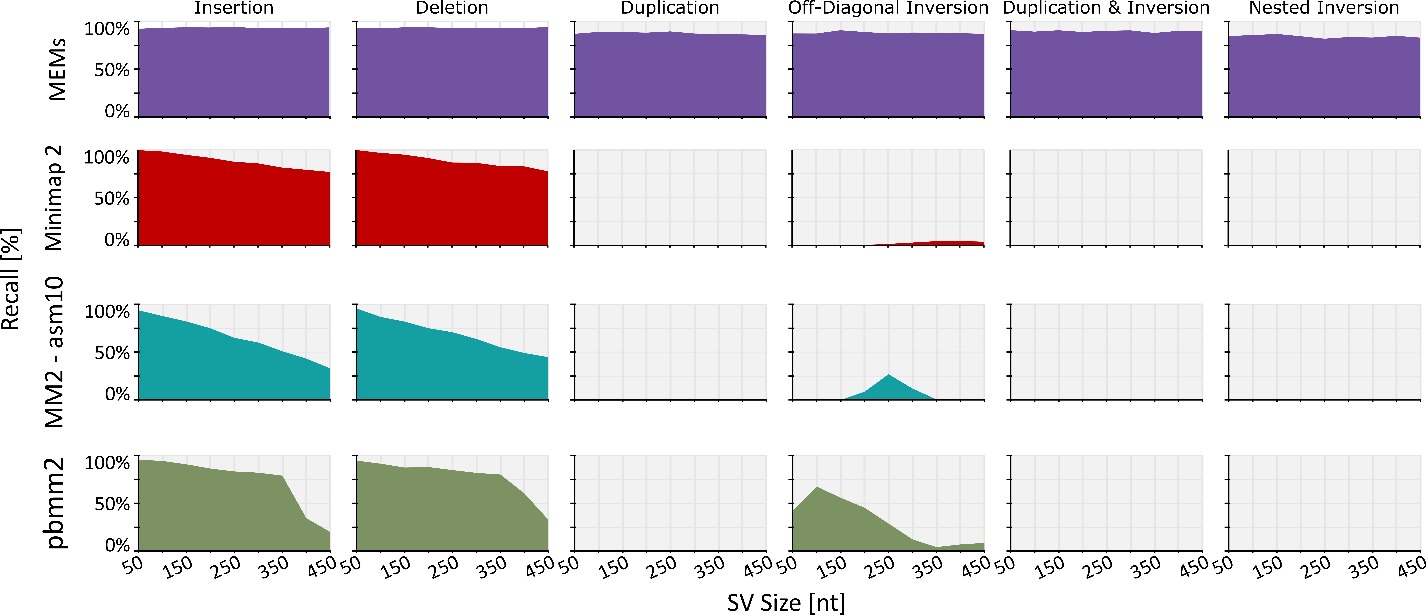


**Figure S6.** Using the same benchmarking as used for Fig. 2 B) in the main manuscript, we perform an additional analysis using minimap2 with the asm10 presetting as well as pbmm2. Here we analyze queries with simulated sequencing errors. pbmm2 is a wrapper script for minimap2 that utilizes presettings customized by Pacific Bioscience. The diagrams demonstrate that, when compared to minimap2 using the presetting used for the benchmarking in the main manuscript, the modified presettings have both positive as well as negative impact to the recall rates, while maintaining the overall pattern. The alignments produced by minimap2 still conceal more complex variants such as a duplication, duplication & inversion, and nested inversions.

## Analyzing smaller and larger queries


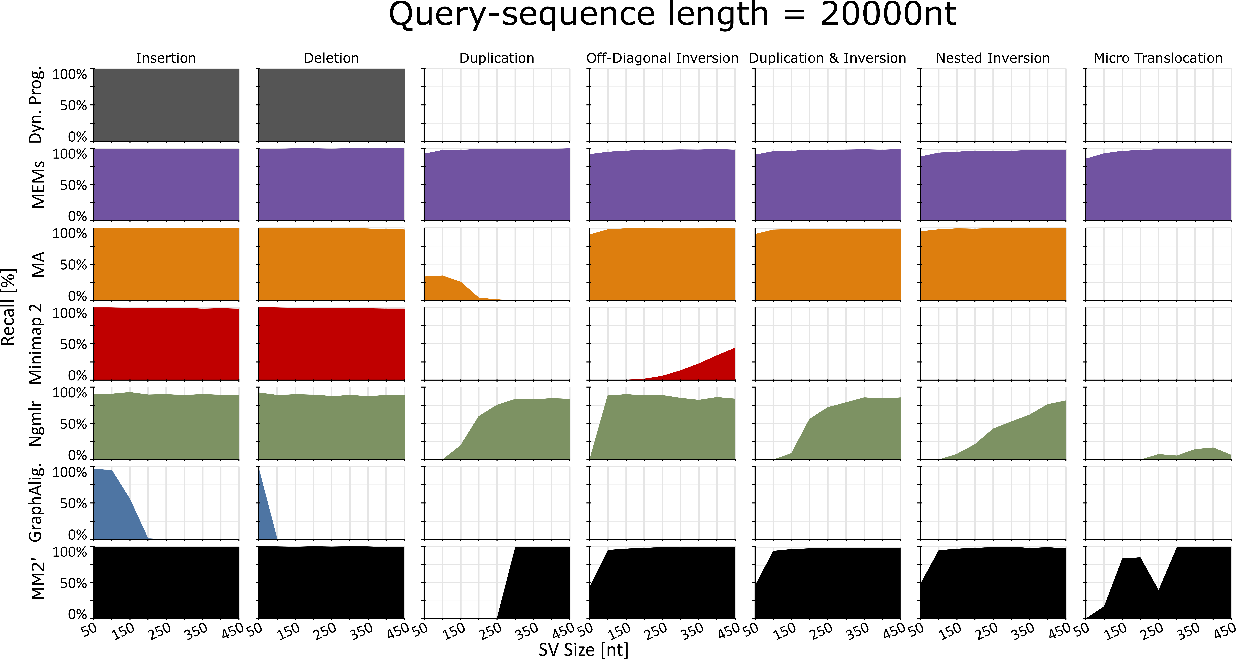


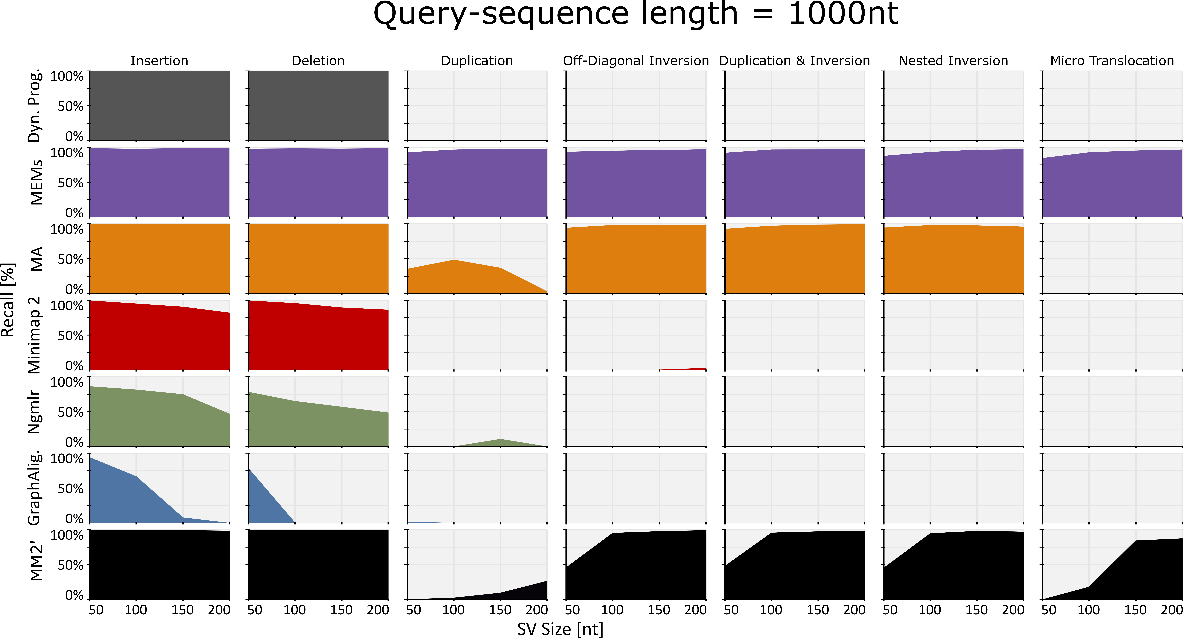


**Figure S7.** Next, we analyze if the query length impacts the discovery of the exemplary genomic rearrangements. For this purpose, we repeat the analysis described in the main text (benchmarking shown in Fig. 2) with the query the lengths 1,000nt (0,5 x 2000nt) and 20,000nt (10 x 2000nt) and sequencer error-free queries. (2000nt is the query length used in the main manuscript). Please note that for the 1,000nt long queries, SV sizes larger than 200nt were not computed, due to the size constraints of the queries. While most techniques tend to improve regarding the recall rate for longer queries and worsen for smaller ones, the overall trend along the y-axis does not change: SV sizes and types with low ($<$10%) and high ($>$90%) recall rates are the same for all query sizes. Among the aligners, NGMLR benefits most from longer query sizes.
