# Supplementary material for: MSV: a modular structural variant caller that reveals nested and complex rearrangements by unifying breakends inferred directly from reads
Source: Genome Biol. 2023 Jul 17;24:170. doi: 10.1186/s13059-023-03009-5 (PMC10351204; doi:10.1186/s13059-023-03009-5)
Supplement: Supplementary file 7 — Additional file 7. Computation of ground truth entries and evaluation of 100nt long Illumina as well as Oxford Nanopore reads. Contains Fig. S8 and S9. [file 13059_2023_3009_MOESM7_ESM.docx]

# Additional file 7: Computation of ground truth entries and evaluation of 100nt long Illumina as well as Oxford Nanopore reads

The computation of ground truth entries happens by applying the entry creation scheme described in the methods section with the sequenced genome as one single error-free read. It consists of the following three steps:

1. Obtain all MEMs using Minimizers and occurrence filtering as described in the main text.

(see “Incorporating genome repetitiveness and sequencer errors into SV calling” in the results section.)

1. Apply an overlap elimination on the MEMs as described in the main text.

(see “Incorporating genome repetitiveness and sequencer errors into SV calling” in the results section.)

1. Turn the MEMs into matrix entries as described in the main text.

(see “SV calling using raw MEMs” in the results section.)

The matrix entries resulting from the final step represent the ground truth. Because the sequenced genome acts as one long perfect read, additional entry processing (e.g. fuzzy inference of edges, computing clusters by merging overlapping entry-areas and approximating true entry locations) is not required here.

For the genome reconstruction process (see Additional file 14), we additionally memorize the visiting order of the matrix entries in a traversal (through the graph corresponding to the matrix) that delivers the sequenced genome. This visiting order is equivalent to the occurrence order of the corresponding MEMs on the sequenced genome.

In addition to analyzing 250nt-long simulated Illumina reads in Fig. 5 of the manuscript, we here also examine 100nt-long simulated reads. For 100nt reads, we observe an improved performance of Manta, while the performance of Gridss and our approach remains mostly unchanged.


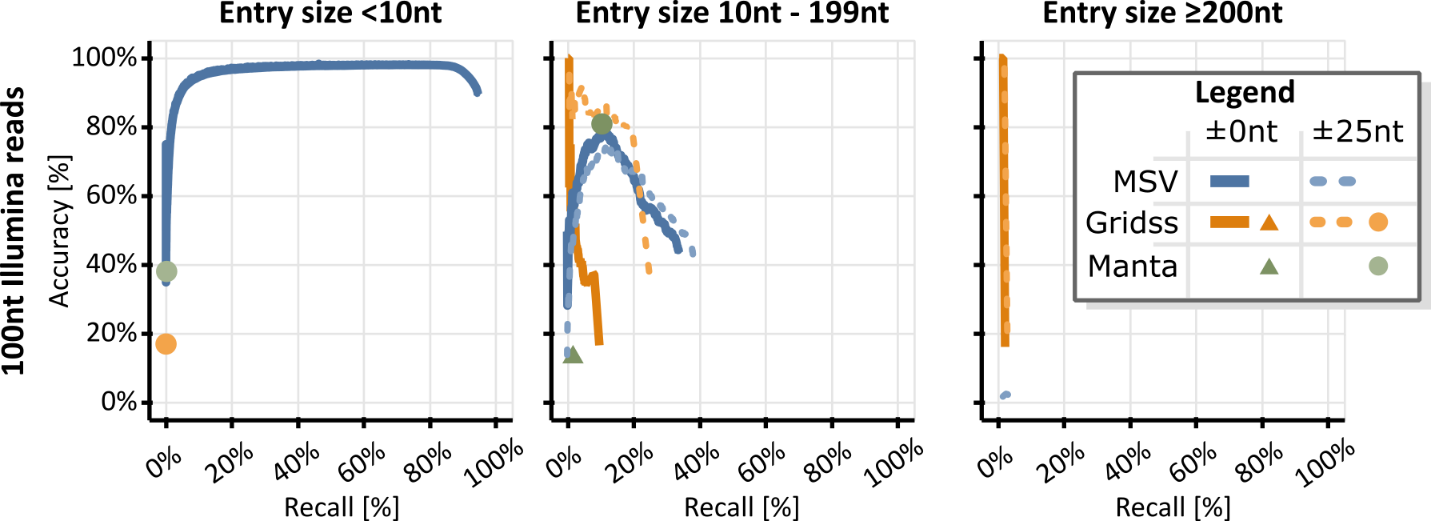


**Figure S8.** Analysis of 100nt long Illumina reads.

We perform the benchmarking analysis of Fig. 5 of the main manuscript using simulated Oxford Nanopore reads. To generate the simulated reads, we rely on SURVIVOR (https://github.com/fritzsedlazeck/SURVIVOR) using the NA12878 nanopore error profile. When compared to the curves for simulated PacBio reads in Figure 5 of the main manuscript, the curves below reveal the following trends: For smaller SVs (<10nt), the high error rate in Oxford Nanopore reads has a moderate impact on recall and accuracy rates. However, for larger structural variations, the high degree of read errors in Oxford Nanopore reads introduces increasing noise in the adjacency matrix, resulting in decreased recall and accuracy rates.


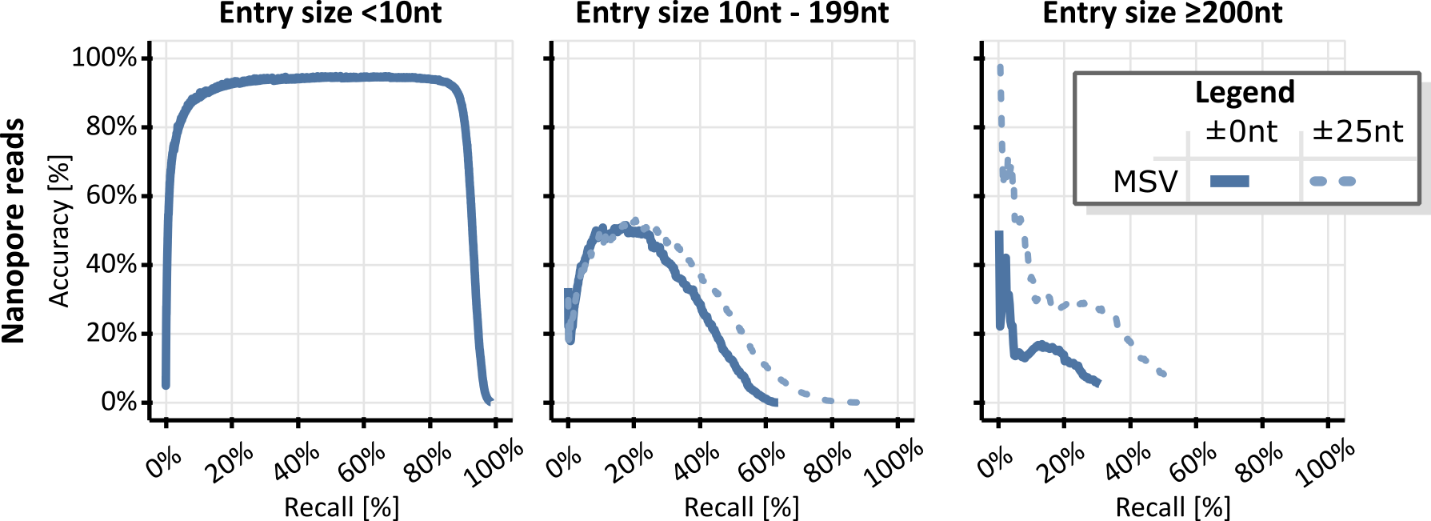


**Figure S9.** Analysis of Nanopore reads.
