# Supplementary material for: MSV: a modular structural variant caller that reveals nested and complex rearrangements by unifying breakends inferred directly from reads
Source: Genome Biol. 2023 Jul 17;24:170. doi: 10.1186/s13059-023-03009-5 (PMC10351204; doi:10.1186/s13059-023-03009-5)
Supplement: Supplementary file 8 — Additional file 8. Reconstructing sequenced genomes from graphs. Contains Fig. S10. [file 13059_2023_3009_MOESM8_ESM.docx]

# Additional file 8: Reconstructing sequenced genomes from graphs

**Figure S10.** Reconstruction of a sequenced genome from a reference genome.

The figure visualizes an example of the reconstruction of a sequenced genome $S=UWYXIZ$ from a reference genome $UVWXYZ$. The reconstruction happens via a single error-free read spanning $S$ (subfigure C1) and via a set $X$ of simulated long reads (PacBio) and short reads (Illumina) for $S$ (subfigure C2). **A)** shows the third quadrant of the two adjacency matrices $M_{S}$and $M_{X}$ for C1) and C2) in combined form, respectively. Here the red entries belong to $M_{S}$ and the blue entries belong to $M_{X}$. $\left( a,a^{'} \right),(b,b^{'})$ and $(d,d^{'})$ represent pairs of spatially closest neighbors in $M_{X}$ and $M_{S}$. The entry $c\in M_{S}$ does not have a corresponding entry in $M_{X}$. Similarly, the entry $g^{'}\in M_{X}$ is without a partner in $M_{S}$. Subfigure **B)** displays the graphs $G_{S}$ and $G_{X}$ for $M_{S}$ and $M_{X}$ in combined form. (Red edges are part of $G_{S}$ and belong to $M_{S}$, while blue edges are part of $G_{X}$ and belong to $M_{X}$.) Here the weight $I$ of the edges $d$ and $d'$ represents a sequence not occurring on the reference genome that is inserted whenever the reconstruction passes through $d$ or $d'$. The tables in **C1)** and **C2)** list the elements of the sets $T_{S}$ and $T_{X}$, respectively. $T_{S}$ and $T_{X}$ describe traversals through their respective graphs, as explained in the main text. Additionally, the tables in C1) and C2) annotate the origin and destination of each row’s edge. The genomes that are reconstructed from the tables are shown to their right. The tables additionally annotate matching (green lines) and non-matching (red lines) connections between the ‘origin’ and ‘destination’ entries of consecutive rows. If the origin of the next edge is not reachable (the wrong direction considering the current strand), no reference sequence is used in reconstruction between the edges. This is the case for $a^{'}\to b'$ and $b^{'}\to d'$ in the above example. Such a correction is necessary to ensure that edges that are slightly misplaced do not break the traversal. Inserted sequences are always reconstructed.
