# Supplementary material for: MSV: a modular structural variant caller that reveals nested and complex rearrangements by unifying breakends inferred directly from reads
Source: Genome Biol. 2023 Jul 17;24:170. doi: 10.1186/s13059-023-03009-5 (PMC10351204; doi:10.1186/s13059-023-03009-5)
Supplement: Supplementary file 9 — Additional file 9. The sentinel vertex. Contains Fig. S11 and Table S1. [file 13059_2023_3009_MOESM9_ESM.docx]

# Additional file 9: The sentinel vertex

**Figure S11. A)** displays a sequenced genome $AIB$ and a reference genome $AB$. $I$ is assumed to be a long insertion that is not fully enclosed by any read. **B)** shows four reads that partially cover $I$ and their genome mapping graph. The two reads $r1$, $r2$ originate from the forward strand, while the remaining two reads $r3$, $r4$ originate from the reverse strand of the sequenced genome. The reads $r1$, $r3$ cover the beginning of $I$, while the reads $r2$, $r4$ cover the end of $I$. All four reads create an edge that connects to the sentinel vertex or its mate (labeled $NIL$ and $\tilde{NIL}$ in the genome mapping graph). **C)** presents the unfolded adjacency matrix for the genome mapping graph in B). Matrix entries without black outlines are implicit (see methods section of main text). Red crosses mark the positions of entries for reads that would fully enclose the insertion. These crosses are in line (either horizontally or vertically) with the entries that connect to sentinel vertices (because their origin or destination vertices are equal) and they can be used for merging these entries. **D)** Forward strand and reverse strand are unified by applying our folding scheme to the adjacency matrix (the methods section of the main text describes this folding scheme). Because of this folding, there are no entries in the $NIL$ column on the right. For applying our clustering to sentinel connecting entries, the sentinel’s row is placed far above the remaining matrix. This prevents clusters of sentinel entries from interfering with clusters of regular entries.

In the following, our approach is extended by the concept of the sentinel vertex for coping with long insertions:

### Creation of sentinel entries in the adjacency matrix

The adjacency matrix is extended by a column and row labeled $NIL$ as well as a column and row labeled $\tilde{NIL}$ for the sentinel vertex and its mate. These inserted rows and columns are placed in central locations of the unfolded adjacency matrix as shown in Fig. S10 C). In the folded matrix, the sentinels correspond to the topmost row and rightmost column.

Let $\left( q,r,l,k \right)$ be a seed with the beginnings $q$ and $r$ on a query $Q$ (read) and reference (genome) as well as a length $l$ and a strand information $k\in\{F,R\}$, where $F$ and $R$ correspond to forward and reverse strand, respectively. Further, let $d$ be a given minimal distance (threshold) that triggers the connection to a sentinel. Sentinel related matrix entries are created as follows:

| **conditions** | | | **outcome** |
| --- | --- | --- | --- |
| **strand information (**$\boldsymbol{k}$**)** | **first or last seed on read** | **distance to end of read** | **entry in adjacency matrix** |
| $F$ | first  (smallest $q$) | $q\geq d$ | $(NIL, r)$ |
| $R$ | first  (smallest $q$) | $q\geq d$ | $(\tilde{NIL}, r+l)$ |
| $F$ | last  (largest $q+l$) | $q+l\leq\left\vert Q \right\vert-d$ | $(r+l,NIL)$ |
| $R$ | last  (largest $q+l$) | $q+l\leq\left\vert Q \right\vert-d$ | $(r,\tilde{NIL})$ |

**Table S1.** Sentinel related matrix entries.

By applying the matrix-folding scheme described in the main text, sentinel entries from forward and reverse strand reads are unified. The distance $d$ must be chosen according to the specific characteristics of a set of reads as e.g. the error-rate of the sequencer and average length of the reads. In the context of our evaluation of yeast genomes, we use a distance $d=50$ for CCS PacBio reads and $d=\infty$ for Illumina reads, where $\infty$ disables the creation of sentinel connecting entries.

### Clustering of sentinel connecting entries

The matrix entries’ clustering described in the methods sections exploits the spatial locality of the vertices (corresponding to the entries) on the reference genome. This spatial locality is void between the sentinels and all other vertices. Therefore, clusters of sentinel connecting entries are not allowed to overlap with clusters of regular entries. As shown in Fig. S10 D), we separate both types of clusters by inserting a void space between them in the adjacency matrix.

### Merging of sentinel connecting entries with regular adjacency matrix entries

In the following, Let $X$ be the set of all reads that cover some section of an insertion $I$. We split the set $X$ into three subsets $X_{Full}$, $X_{In}$, $X_{Out}$ as follows: $X_{Full}$ comprises all reads in $X$ that fully enclose the insertion $I$. $X_{In}$ consists of all reads in $X$ that cover the beginning of $I$ but do not fully enclose $I$. Accordingly, $X_{Out}$ keeps all reads of $X$ that cover the end of $I$ but do not fully enclose $I$.

For all reads in $X_{Full}$, the resulting entries for $I$ are regular entries that are created via the breakends of two seeds. In contrast, the reads in $X_{In}$ and $X_{Out}$ connect to the sentinels for $I$. However, the origin of the entries in $X_{In}$ matches the origin of the entries in $X_{Full}$ and the destination of the entries in $X_{Out}$ matches the destination of the entries in $X_{Full}$. Via these relationships, it is possible to merge the entries of all three subsets to one single matrix entry. In the context of a matrix entry’s scoring, this merging is exploited for measuring its number of supporting reads.
