# Supplementary material for: MSV: a modular structural variant caller that reveals nested and complex rearrangements by unifying breakends inferred directly from reads
Source: Genome Biol. 2023 Jul 17;24:170. doi: 10.1186/s13059-023-03009-5 (PMC10351204; doi:10.1186/s13059-023-03009-5)
Supplement: Supplementary file 10 — Additional file 10. Ambiguities inherent to basic SV. Contains Fig. S12. [file 13059_2023_3009_MOESM10_ESM.docx]

# Additional file 10: Ambiguities inherent to basic SV

**Figure S12.** The figure shows examples for the ambiguities mentioned in the discussion section and Fig. 5 of the main text. **A)** is a copy of Fig. 5 with additional annotations that indicate the locations of the cases B) to D) regarding the remainder of the above figure.

Subfigure **B)** shows that even in simple cases with isolated basic SV, the history (order of occurrence) cannot be reconstructed from the sequenced genome.

**C)** The left and right sequenced genome have identical associated breakends (center column) on the reference genome. However, the left sequenced genome has two duplications of $B$ while the right sequenced genome comprises one duplication merely. Our approach catches the equivalence of the associated breakends via a corresponding equivalence in the adjacency matrix, while the difference in the number of occurrence of $B$ corresponds to two different graph traversals. The separation of adjacency matrix (breakend associations) and traversal (order of breakend associations on the sequenced genome) is mandatory due to the limited size of real-world reads (see subfigure E)).

**D)** In the top row, the sequenced genome $ACBD$ is created using two different sets of basic SV from the reference genome $ABCD$. Accordingly, in the bottom row, a duplication and an inversion on the section $B$ are used for creating two different sequenced genomes. Notably, the basic SVs are applied in the same order for both outcomes.

**E)** Each read that does not span the full genome delivers a partial graph traversal merely. Often, these partial graph traversals cannot be combined into a full traversal. Particularly with short reads, such a combination is often impossible in real-world scenarios.
