# Supplementary material for: MSV: a modular structural variant caller that reveals nested and complex rearrangements by unifying breakends inferred directly from reads
Source: Genome Biol. 2023 Jul 17;24:170. doi: 10.1186/s13059-023-03009-5 (PMC10351204; doi:10.1186/s13059-023-03009-5)
Supplement: Supplementary file 11 — Additional file 11. Detailed matrix folding of Fig. 7 in the methodssection. Contains Fig. S13. [file 13059_2023_3009_MOESM11_ESM.docx]

# Additional file 11: Detailed matrix folding of Fig. 7 in the methods section

**Figure S13.** The figure shows the detailed folding of the adjacency matrix in Fig. 6 E) in the main text.
