# Supplementary material for: MSV: a modular structural variant caller that reveals nested and complex rearrangements by unifying breakends inferred directly from reads
Source: Genome Biol. 2023 Jul 17;24:170. doi: 10.1186/s13059-023-03009-5 (PMC10351204; doi:10.1186/s13059-023-03009-5)
Supplement: Supplementary file 12 — Additional file 12. Detailed description of overlap-elimination. Contains Fig. S14. [file 13059_2023_3009_MOESM12_ESM.docx]

# Additional file 12: Detailed description of overlap-elimination

**Figure S14.** Overlap elimination.

Figure 3 D) of the results section displays a situation, where two MEMs overlap on the y-axis of a diagrammatic dot-plot. “Computing MEMs for a read via reseeding” of the methods section introduces an overlap elimination technique for managing such overlaps. Subfigure S13 **A)** and **B)** illustrate the cutting scheme described there. The labels $m_{1}$, $m_{2}$ and $p_{cut}$ correspond to the two MEMs and to the central cutting location mentioned in the main text, respectively.

If the outer ends of large-scale SV belong to repetitive regions on the reference genome, the overlaps visualized in **C)** between two clusters of MEMs occur. Here, an application of the overlap elimination on MEMs removes all overlaps but connects the repetitive regions with several entries in both directions. However, the repetitive regions should be connected by one entry merely. For solving this problem, overlap elimination is applied to MEM-clusters as well. For MEM-cluster creation, we rely on Strips of Consideration [22]. Let $C$ denote a cluster and $\left( q,r,l \right)$ a MEM in $C$, where $q,r$ and $l$ are the start position on query, the start position on reference and length of the MEM, respectively. The read interval of $C$ is defined as

$$I_{C}:=\left[ \min\left\{ q|\left( q,r,l \right)\in C \right\},\max\left\{ q+l|\left( q,r,l \right)\in C \right\} \right).$$

The overlap elimination scheme for MEMs as described in the main text is applied to MEM-clusters using their respective read intervals. **D)** visualizes one special case for enclosed intervals. If the enclosing MEM-cluster and the enclosed cluster do not comprise overlapping seeds, both clusters are kept.
