# Supplementary material for: MSV: a modular structural variant caller that reveals nested and complex rearrangements by unifying breakends inferred directly from reads
Source: Genome Biol. 2023 Jul 17;24:170. doi: 10.1186/s13059-023-03009-5 (PMC10351204; doi:10.1186/s13059-023-03009-5)
Supplement: Supplementary file 13 — Additional file 13. Fuzzy inference of edges from MEMs. Contains Fig. S15. [file 13059_2023_3009_MOESM13_ESM.docx]

# Additional file 13: Fuzzy inference of edges from MEMs

**Figure S15.** The impact of sequencing errors on matrix entries. **A)** In $r1$, two sequencing errors cause the MEMs $s1.1$ and $s1.4$ to break prematurely regarding the breakend pair that is visualized as an orange line. As a result, the two short MEMs $s1.2$ and $s1.3$appear. We assume that these two MEMs get lost during the occurrence filtering because of their small size. Due to the absence of these two MEMs, we get the entry $e$ in B). In $r2$, the first sequencing error causes $s2.1$ to erroneously extend over the breakend on the sequenced genome, while the second sequencing error causes a premature breaking of $s2.2$. The two MEMs $s2.1$ and $s2.2$ create the entry $e'$ in B). **B)** shows the entry areas in the folded adjacency matrix for the entries $e$ and $e'$ as well as three more fictional entries. Entry areas are visualized as light blue squares. Further, the subfigure indicates the location of the true entry via a red box (matrix entry). The distances $\sigma$ and $f$ are expressed with respect to the entry $e$. The gray shaded sidebars labeled $X$ and $Y$ display the two sets used for the approximation of the true entry’s location. In this context, the red lines in the sidebars visualize the true entry location according to our proposed percentile scheme (5% to 95%).
