# Supplementary material for: MSV: a modular structural variant caller that reveals nested and complex rearrangements by unifying breakends inferred directly from reads
Source: Genome Biol. 2023 Jul 17;24:170. doi: 10.1186/s13059-023-03009-5 (PMC10351204; doi:10.1186/s13059-023-03009-5)
Supplement: Supplementary file 14 — Additional file 14. Line-sweep based clustering of entry-areas. Contains Fig. S16 and S17. [file 13059_2023_3009_MOESM14_ESM.docx]

# Additional file 14: Line-sweep based clustering of entry-areas

**Figure S16.** Line-sweep based clustering of entry-areas

Practically, we store entry-areas as a list of rectangles, where the same rectangle can occur multiple times. By sorting this list followed by a line-sweep, we can identify overlapping entry-areas in the matrix. This line-sweep based clustering starts with an inexact sweep that separates the whole dataset into smaller inexact clusters. Here, an inexact cluster may be comprised of several actual clusters (i.e. sets of rectangles that actually overlap), but actual clusters always belong to a single inexact cluster. Given an inexact cluster, we subsequentially compute the actual clusters within it using an exact line-sweep. Theoretically, the exact line-sweep is capable of computing the exact clusters directly from the complete dataset; however, this would increase memory requirements and runtime tremendously.

## The exact algorithm

We start by explaining the exact line-sweep: The algorithm sweeps horizontally (along the x-axis) over a set of $n$ entry areas. To store which areas overlap on the y-axis, we use two $n$ sized vectors, called counting vector $V_{c}$ and pointer vector $V_{p}.$ $V_{c}$ and $V_{p}$ are initialized with zeros and null pointers, respectively. During the line-sweep,$V_{c}$’s purpose is to hold the number of overlapping entry areas for each y-position, while the pointers in $V_{p}$shall point to the clusters of entry areas that occupy the respective y-positions. To map the y-values of all entry areas to the indices of the two vectors, we apply y-squeezing to all entry areas. y-squeezing is achieved by creating a sorted list of all y-values (top and bottom of all entry-areas) and using each y-value’s index in the sorted list as the index for $V_{c}$ and $V_{p}$. The line sweep iterates over the x-values of the start and end-points of all entry areas in ascending order. At the start point of each area, we merge this area with all overlapping clusters (stored in $V_{p}$). At the end-point of each area, we check (using $V_{c}$) if we can remove the area’s cluster from $V_{p}$. In detail, this is performed as follows:

- Whenever the line sweep stops at the start position of an entry area $e=\left( e_{top},e_{bottom} \right)$, we increase all counters in $V_{c}\left[ e_{top},e_{bottom} \right]$. Further, let $C$ be the set of clusters pointed to in $V_{p}\left[ e_{top},e_{bottom} \right]$. We create a new set $S$, that contains all entries in $C$ as well as $e$. Let $P$ be the set of pointers in $V_{p}$ that point to any cluster in $C$. (Note that $P$ contains all pointers in $V_{p}\left[ e_{top},e_{bottom} \right]$, but may contain further pointers.) All pointers in $P$ are redirected to $S$. This redirection does not require a scan over the full vector, since we memorize the maximal y-axis interval of each cluster. However, before redirecting a pointer in the y-axis interval of a cluster, we need to verify that the pointer is not pointing to another cluster, as a cluster can be hollow.
- Whenever the line-sweep stops at the end position of an entry area$e=\left( e_{top},e_{bottom} \right)$, we decrease all entries in $V_{c}\left[ e_{top},e_{bottom} \right]$. For each value in $V_{c}$ that becomes zero, we set the respective pointer in $V_{p}$ to null. We detect if this step removes the last pointer to a cluster by memorizing the number of active pointers for each cluster. Any cluster that becomes pointer-less is saved as the output of the line sweep.

## The inexact (heuristic) algorithm

**Figure S17.** Compressed pointer vector.

The exact algorithm requires memory relative to the size of $V_{c}$ and $V_{p}$, which is too large for big genomes. Hence, the inexact line sweep uses a compressed form of the pointer vector and drops the counting vector. This compression is achieved by having each counter represent an interval instead of a single point on the y-axis. However, there is a high density of entries just above the diagonal, where a compressed vector would lead to the creation of a single genome-spanning cluster. To avoid this genome-spanning cluster, we do not compress $V_{p}$ in this area of high density. Since the area of high-density moves along the diagonal (i.e. upwards on the y-axis) during the line sweep, we perform a coordinate system transformation on the y-coordinates of all entries. With the transformed y-coordinates, the high-density area remains in the same interval for the entire line-sweep. We compute the new y-coordinates ${e'}_{top}$ and ${e'}_{bottom}$ for each entry area $e$ as follows:

$${e'}_{top}=e_{top}-e_{left}$$

$${e'}_{bottom}=e_{bottom}-e_{right}$$

Note that this transformation turns all rectangles into parallelograms in the original coordinate system. By placing all parallelograms so that they surround their original rectangles, we ensure that exact clusters of rectangles end up in the same inexact cluster.

Instead of using $V_{c}$ to track the exact extension of each set on the y-axis, we use the memorized maximal y-axis interval as well as a counter of open entry areas for each set. This saves us from adjusting $V_{c}$ but leads to clusters casting “shadows” to their right. (This shadow ends at the endpoint of the rightmost parallelograms in the cluster.) Clusters that are erroneously joined due to the “shadows” or the coordinate transformation are separated again during the exact line-sweep.
