# Supplementary material for: MSV: a modular structural variant caller that reveals nested and complex rearrangements by unifying breakends inferred directly from reads
Source: Genome Biol. 2023 Jul 17;24:170. doi: 10.1186/s13059-023-03009-5 (PMC10351204; doi:10.1186/s13059-023-03009-5)
Supplement: Supplementary file 15 — Additional file 15. Review history. [file 13059_2023_3009_MOESM15_ESM.docx]

**1^st^ round**

**Reviewer 1**

The manuscript addressed an important problem related to SV calling and ambiguity of complex genome rearrangements. I think it has a lot of value for the community to acknowledge this problem really exists and offer a solution. The paper discusses some of the limitations of their method and explores possible ways to address them. The approach is novel and highlights the problems of current SV detection (such as ambiguity in positions (can be additionally highlighted) and ambiguity in composition). While the approach is limited at the moment, I found this research valuable.

I have several comments, mostly minor, that I think needs to be address:

Major comments:

1) While there is a bit of discussion about this in the paper, the applicability of the solution to the human genome needs to be discussed in more detail. How common and challenging complex SVs are in human genomes? What are the limitations of the method?

2) While Sniffles is a decent tool for long-reads, Delly is not the best choice for short-read SV calling. I'd instead suggest using Manta and Gridss which are showed in several recent benchmark papers to be more accurate.

Minor comments:

3) "A set of atomic SVs can describe multiple reorganizations" - this is true, but if a tool is aiming to reconstruct nested SVs it should be able to order overlapping events. Could you provide examples of popular tools that have such problems?

4) Figure 2B is messy, too many lines are overlapping and it is hard to understand where to find them. I need 300% zoom in to distinguish something.

5) Figure 2A, part (dup + inv) is probably incorrect, also brackets for (nested inversion) are not correct

6) "Each point represents an average measurement for 10,000 reads of length 2,000 nt." - Only possible to understand after reading the main text. There is a minor problem that not all reads are 2kbp in length. Also, I'm not sure if reads is a good name for these sequences

7) Please, introduce MEMs in a more intuitive way, I understood how and why they work only after a deep thought. Also, it somehow explains the set of SVs for Figure 2.

8) In the conclusion. This deficiency of current approaches leads to the exclusion of nested and complex variants in many contemporary studies (as e.g. cites) ◊ the specific citations are missing

**Reviewer 2**

In this manuscript, Schmidt and Kutzner describe ambiguities in current structural variant (SV) assessing tools and present rationale for a MEM-based (Maximal Exact Matches) genome-mapping graph model to resolve those ambiguities. Schmidt and Kutzner highlight flaws in current SV callers like Sniffles and Delly that rely on split-read alignment tools. They show that, other than MEM-based aligners, most alignment tools do not accurately identify complex SVs. Aligning sequences to graphs is a newer field and thus Schmidt and Kutzner propose a novel technique for computing genome-mapping graphs using MEMs. The algorithm developed by Schmidt and Kutzner is an adjacency matrix-based model that can cope with complex and arbitrarily nested SVs. While they propose a novel model, the majority of the presented work is based on theoretical examples and simulated data. The authors also use the analysis of two wild yeast genomes. The suitability of the approach is not tested on any other real dataset, nor on any large genome. These are not appropriate examples to use to assess SV detection in mammalian genomes (where we see SVs the most).
Other specific comments are listed below:

Major comments:
* The manuscript is very narrow in its focus and is not "of utility to a wide audience". While the authors propose a novel SV calling algorithm, the bulk of this Methods paper reads more like a review article, focusing primarily on the weaknesses of current SV calling methods, followed by the rationale for developing their model and an in-depth description and use of their algorithm. Genome Biology submission guidelines state that "Method articles should describe novel methods that are shown to be a clear advance over existing state-of-the-art methods in a side-by-side demonstration". This manuscript fails to do so. While it shows that MEM-based aligners are better at calling complex Structural Variants, the authors fail to compare their novel model to other graph-based aligners (such as e.g., GraphAligner, https://doi.org/10.1186/s13059-020-02157-2). This comparison will be necessary for assessing whether a MEM-based genome-mapping graph model is superior to other models currently available
* There are serious concerns about the way the text has been presented to the readership. Many sections of the study are impenetrable to the general biologist. Perhaps adding additional sub-sections and stating more clearly the aims of each section would improve the paper. It does not help that the authors explain their approach in the results sections (only partially) and frequently refer to the Methods section and Supplementary Notes; never explaining their approach fully in one place.
* The authors do not clarify why they have chosen certain parameters for their analyses. For example, on pages 6-7 the authors conduct an analysis using sequence read lengths of 2000 bp. There is no explanation why such a read length has been chosen, especially since it does not correspond to the read length of any currently-used sequencing technology that I am aware of. A read length closer to what is currently produced by current sequencing technologies might be a more suitable and informative choice for this parameter. Additionally, for the majority of the analysis, error-free sequence data is used,, again not applicable to real data where a percent of sequencing errors are indeed inherent.

Minor comments:
* The title of the paper is not very informative. Perhaps the authors could consider changing the title to something that better describes the content/achievements of the manuscript.
* What is the meaning of 'atomic SV'. It is not a commonly used term in biology and it would be good to define it in the beginning.
* There are many alignment-based, assembly-based and meta-SV callers for both short and long reads data. Can the authors please specify why they specifically chose Sniffles and Delly for their study?
* The figure legends need to be clearer. i.e. Fig 1 bottom panel "Real world caller output" what do the number 3 and 5 followed by INV-3 and INV-5 indicate? What kind of data was used for Fig 2. Was it simulated human data? Can the authors please clarify this?
* In ref 11, SV-caller is a workflow that integrates 4 tools (Manta/Lumpy/Delly/GRIDSS). In this manuscript, the SV-caller is not a specific term. Instead, it indicates multiple callers in different sections. It is confusing and I would suggest removing the hyphen in between.
* The citations for the figures are not organized in numeric order and are referenced as Fig 1, Fig 3, Fig 6, Fig2b, Fig 2, etc.

**Authors’ response**

Reviewer 1

**Comment 1:** While there is a bit of discussion about this in the paper, the applicability of the solution to the human genome needs to be discussed in more detail. How common and challenging complex SVs are in human genomes? What are the limitations of the method?

**Fixed:** We extended and reorganized the discussion section so that the applicability of our approach to the human genome gets extra attention and a more exposed position. For this purpose, we changed the discussion section’s original order of paragraphs from:

1. Ambiguity contextualization, 2. Genome repetitiveness, 3. Applicability to human genome

to:

1. Applicability to human genome, 2. Genome repetitiveness, 3. Ambiguity contextualization.

The new layout of the discussion section allows a better linkage between the three topics of the section. Now, we start with the applicability of our approach to the human genome and can lead the reader to a major problem in the context of the human genome’s analysis: the genome’s repetitiveness. In the repetitiveness paragraph, we explain the limits of our approach or generally, all approaches for SV calling. The paragraph on repetitiveness, in turn, leads the reader to ambiguity-related problems that are discussed at the end of the discussion section. In this context, we now cite two recent publications on patterns of somatic structural variations in the human genome. This shall support answering the referee’s subquestion “How common and challenging complex SVs are in human genomes?”

**Comment 2:** While Sniffles is a decent tool for long-reads, Delly is not the best choice for short-read SV calling. I'd instead suggest using Manta and Gridss which are showed in several recent benchmark papers to be more accurate.

**Fixed:** We now analyze both Gridss and Manta. Further, we now cite several recent benchmark papers that show that these tools are state-of-the-art.

**Minor comments:**

**Comment 3:** "A set of atomic SVs can describe multiple reorganizations" - this is true, but if a tool is aiming to reconstruct nested SVs it should be able to order overlapping events. Could you provide examples of popular tools that have such problems?

**Response:** We are not aware of tools that order overlapping basic SV (we have renamed atomic SV to basic SV due to the referee’s comments below). In the manuscript, we state that, to our knowledge, state-of-the-art calling techniques deal with nested rearrangements as follows: they define specific breakend/breakpoint patterns and then analyze matches (inferred from alignments) to these patterns. However, SV callers that call breakends merely (e.g. Gridss) do not need/perform this ordering.

**Comment 4:** Figure 2B is messy, too many lines are overlapping and it is hard to understand where to find them. I need 300% zoom in to distinguish something.

**Fixed:** To fix this, we experimented with several forms of visualizations incl. bar diagrams. Finally, we decided to go for the “area plots” now visible in Fig. 2B. These plots allow the reader to quickly recognize the allover “rediscovery behavior” of the various approaches/aligners while still being able to compare them due to the vertical alignment of the plots. As requested by referee #2, the figure now additionally comprises curves for GraphAligner (2).

**Comment 5:** Figure 2A, part (dup + inv) is probably incorrect, also brackets for (nested inversion) are not correct.

**Fixed:** Thank you for pointing this out, this was absolutely an oversight on our end! We rotated the appropriate genome section and changed the size of the brackets.

**Comment 6:** "Each point represents an average measurement for 10,000 reads of length 2,000 nt." – Only possible to understand after reading the main text.

**Fixed:** We now report the sizes of all involved sequences visually on the axes of the diagrammatic dot-plots in Fig. 2A. Therefore, the 2000nt query length (reads have been renamed to queries to address the issue below) is now directly indicated on the y-axis of the dot plots.

**Comment 7:** There is a minor problem that not all reads are 2kbp in length. Also, I'm not sure if reads is a good name for these sequences

**Fixed:** We exchanged the notion “read” by the notion “query” in the context of the benchmarking.  The latter notion originates from aligners and therefore should be more suitable. The specific query (read) size of 2000nt was chosen for giving the benchmarked aligners/techniques a good chance to find the sections before and after the injected SV, which are of size 50nt to 450nt. To prove that the read size is quite irrelevant in the context of the behavior of the benchmarked aligners/techniques, we additionally ran the experiment with 20knt and 1000nt query length and reported the outcome in the supplement. The curves over there show the same characteristics as in Fig. 2B. Finally, we added some justification for using error-free queries in the main text that was missing before.

**Comment 8:** Please, introduce MEMs in a more intuitive way, I understood how and why they work only after a deep thought. Also, it somehow explains the set of SVs for Figure 2.

**Fixed:** On page 8, the main text now comprises: “*Maximal Exact Matches (MEMs) are a particular form of seeds, where seeds are equivalences between a reference genome and a read, typically used by an aligner as the basis for alignment computation. Informally, a MEM is such an equivalence between two sequences (e.g. reference genome and read) that cannot be extended in either direction without encountering a mismatch or the outer end of one (or both) of the sequences.”* Additionally, in Fig. 3B the example’s MEMs are visually indicated.

**Comment 9:** In the conclusion. This deficiency of current approaches leads to the exclusion of nested and complex variants in many contemporary studies (as e.g. cites) ◊ the specific citations are missing.

**Fixed:** We inserted the missing citations.

Reviewer 2

**Comment 1:** The manuscript is very narrow in its focus and is not "of utility to a wide audience".

**Response:** As highlighted in our work, the scheme for reporting SV needs to be changed to advance in the field of SV calling, particularly for nested and complex SV (due to the ambiguities we discuss). This insight goes beyond the approach we developed as it relates to all downstream analyses as well as all other methods that attempt to call complex and nested SV. Further, our work proposes the representation of genomes as skew-symmetric graphs. This kind of genome representation delivers a convenient unification of forward strand and reverse strand and can be used with other forms of genome analyses as well, apart from SV calling. Altogether, we think that the points mentioned above together with our proposed solution are of value to everyone who works with structural variants and beyond.

**Comment 2:** While the authors propose a novel SV calling algorithm, the bulk of this Methods paper reads more like a review article, focusing primarily on the weaknesses of current SV calling methods, followed by the rationale for developing their model and an in-depth description and use of their algorithm. Genome Biology submission guidelines state that "Method articles should describe novel methods that are shown to be a clear advance over existing state-of-the-art methods in a side-by-side demonstration". This manuscript fails to do so.

**Response:**  The referee cites the Genome Biology submission guidelines: “Method articles should A) describe novel methods that B) are shown to be a clear advance over existing state-of-the-art methods in a side-by-side demonstration". Here, we would like to annotate:

A. The methods section of the manuscript describes our novel approach in full detail. Additionally, there is now a short informal description of our approach in the results section as well.

B. In the manuscript, we give several side-by-side comparisons:
1. In Fig. 1 of the manuscript, the state-of-the-art representation scheme for SV is compared to our proposed representation. Here we demonstrate the conceptual advantage of our approach for calling nested SV directly from reads.
2. In Fig. 2 of the manuscript, several state-of-the-art read aligners are compared to the MEM-based approach we use. Here we show that the alignments computed by these aligners can conceal SVs while MEMs do not.
3. In Fig. 5 of the manuscript, we now additionally compare our approach in its entirety to two other SV callers, Gridss and Manta. There, we display the accuracy rate as a function of the recall rate for all competitors and show that our approach outperforms all others. (Sniffles and Delly had to be excluded from this comparison due to their reporting of rearrangements via basic SV and the ambiguities reported for this kind of SV representation in the first part of the results section.)

Referee #2 presumably expects that A) and B) should be presented in a strongly consecutive form, where a presentation of the novel approach is followed by a side-by-side comparison with other state-of-the-art methods. However, we would like to keep the sections in their current order, for the following reasons: B1) and B2) represent two problems that, based on our information, are not common knowledge to the field (referee #1 seems to agree here). We hence deem it necessary to introduce the reader to both problems before presenting a solution to them.

**Comment 3:** While it shows that MEM-based aligners are better at calling complex Structural Variants, the authors fail to compare their novel model to other graph-based aligners (such as e.g., GraphAligner, https://doi.org/10.1186/s13059-020-02157-2). This comparison will be necessary for assessing whether a MEM-based genome-mapping graph model is superior to other models currently available

**(TL;DR) Fixed:** We now analyze GraphAligner too.

**Detailed response:** We suspect a slight confusion here and therefore would like to make some additional remarks: We present a graph-based SV caller, not a graph-based aligner. Our approach relies on the representation of a genome as a skew-symmetric graph and embeds the SV calling into this graph model. The graph is represented via an adjacency matrix that, in turn, is computed using a MEM-based seeding. This seeding is the only resemblance of our model to aligners that typically start the alignment computation with such a seeding step as well. Graph-based aligners (such as GraphAligner) use a graph as a reference, in contrast to our approach and many seed-chain-extend aligners that use a classical assembly as reference. To build such a graph, SVs must be known before the alignment. This is very helpful when looking for specific known SVs that are relevant to some phenotype or disease. However, if SVs are called “de-novo” (as we do here) and therefore variants are not known beforehand, the graph degenerates to a linear sequence. As consequence, GraphAligner loses its main advantage: the graph. We ran GraphAligner with such a degenerated graph and present the results in the main manuscript. GraphAligner’s modest performance reported there has to be understood before this background.

However, our manuscript now comprises an additional comparison with two other SV callers (Gridss and Manta) that hopefully helps with the referee’s remark regarding superiority over other models currently available.

**Comment 4:** There are serious concerns about the way the text has been presented to the readership. Many sections of the study are impenetrable to the general biologist. Perhaps adding additional sub-sections and stating more clearly the aims of each section would improve the paper. It does not help that the authors explain their approach in the results sections (only partially) and frequently refer to the Methods section and Supplementary Notes; never explaining their approach fully in one place.

**Fixed:** We took this comment very seriously and therefore completely rewrote the subsection “SV calling using genome-mapping graphs inferred from MEMs” of the results section. As part of this rewriting, we added Fig. 3. The approach is now informally explained in one place while all the details are still in the methods section. In combination with the other modifications, we hope the readability of the manuscript for the general biologist could be improved significantly.

**Comment 5:** The authors do not clarify why they have chosen certain parameters for their analyses. For example, on pages 6-7 the authors conduct an analysis using sequence read lengths of 2000 bp. There is no explanation why such a read length has been chosen, especially since it does not correspond to the read length of any currently-used sequencing technology that I am aware of. A read length closer to what is currently produced by current sequencing technologies might be a more suitable and informative choice for this parameter. Additionally, for the majority of the analysis, error-free sequence data is used,, again not applicable to real data where a percent of sequencing errors are indeed inherent.

**Fixed:** In the context of the benchmarking shown in Fig. 2, we exchanged the notion “read” by the notion “query” following the request of another referee. The specific query (read) size of 2000nt was chosen for giving the benchmarked aligners/techniques a fair chance to find the sections before and after the injected SV, which are of size 50nt to 450nt. However, to address the referee's worries regarding this specific length, we additionally ran the experiment with 1000nt and 20knt query (read) length and reported the outcome in Supplementary Note 5. The curves over there show the same characteristics as in Fig. 2B. This allows the conclusion that the query (read) length is quite irrelevant in the context of the general behavior (concealing of SV) of the benchmarked aligners/techniques. This is not surprising because, independent of the query size, their strategies for alignment computation always conflict in the same way with a specific rearrangement. E.g. Insertions and Deletions are generally very well detected by almost all aligners/techniques while Duplications pose a major challenge to all of them except for MEMs.

The use of error-free reads in some of the analyses has the background that the evaluated tools/methods shall not be disturbed by other factors as e.g. sequencing errors. These analyses shall show that specific problems (e.g. concealing of SV by alignments) exist already in an idealized world of error-free reads. However, we changed the main text so that this motivation is stated clearer in the manuscript.

**Minor comments:**

**Comment 6:** The title of the paper is not very informative. Perhaps the authors could consider changing the title to something that better describes the content/achievements of the manuscript.

**Fixed:** We have changed the title to: “Structural variant calling: Revealing nested and complex rearrangements by unifying breakends inferred directly from reads” The new title resembles the original title regarding the basic layout but now captures the main achievement of our work.

**Comment 7:** There are many alignment-based, assembly-based and meta-SV callers for both short and long reads data. Can the authors please specify why they specifically chose Sniffles and Delly for their study?

**Fixed:** As a major addition to the original manuscript, we now compare our approach to the two SV callers Gridss (1) and Manta (3) as well. In contrast to Sniffles and Delly, these callers report SV via breakends and could therefore be included in the benchmarking shown in Fig. 5. In this context, we cite several recent benchmark papers that report that Gridss and Manta are state-of-the-art callers, as suggested by referee #1.

**Comment 8:** What is the meaning of 'atomic SV'.  It is not a commonly used term in biology and it would be good to define it in the beginning.

**Fixed:** We changed the notion “atomic SV” to the commonly used notion “basic SV”, which has the same meaning. Additionally, the results section now starts with a definition of the notion “basic SV”.

**Comment 9:** The figure legends need to be clearer. i.e. Fig 1 bottom panel "Real world caller output" what do the number 3 and 5 followed by INV-3 and INV-5 indicate?

**Fixed:** A legend has been added to the figure to explain INV-3 and INV-5. “Real world caller output” has been renamed to “Basic SV calls for 100x coverage, error-free alignments”. Further, we have improved the legends for several other figures.

**Comment 10:** What kind of data was used for Fig 2. Was it simulated human data?

**Fixed:** We now state in the main text as well as the figure legend of Fig. 2 that the human genome assembly GRCh38.p12 is used as a reference and that error-free queries of size 2,000nt were simulated. Further, the outcomes for two additional query sizes (20knt and 1000nt) are in the supplement.

**Comment 11:** In ref 11, SV-caller is a workflow that integrates 4 tools (Manta/Lumpy/Delly/GRIDSS). In this manuscript, the SV-caller is not a specific term. Instead, it indicates multiple callers in different sections. It is confusing and I would suggest removing the hyphen in between.

**Fixed:** We removed the hyphen for eliminating the confusion.

**Comment 12:** The citations for the figures are not organized in numeric order and are referenced as Fig 1, Fig 3, Fig 6, Fig2b, Fig 2, etc.

**Fixed.** We fixed this point, now the citations of the figures are organized in numeric order.

**References**

1.	Cameron DL, Schröder J, Penington JS, Do H, Molania R, Dobrovic A, et al. GRIDSS: sensitive and specific genomic rearrangement detection using positional de Bruijn graph assembly. Genome research. 2017;27(12):2050-60.

2.	Rautiainen M, Marschall T. GraphAligner: rapid and versatile sequence-to-graph alignment. Genome biology. 2020;21(1):1-28.

3.	Chen X, Schulz-Trieglaff O, Shaw R, Barnes B, Schlesinger F, Källberg M, et al. Manta: rapid detection of structural variants and indels for germline and cancer sequencing applications. Bioinformatics. 2015;32(8):1220-2.

**2^nd^ round**

**Reviewer 1**

I don’t have major comments for the paper. I think that this paper is important and timely.

Some parts of the paper still needs better clarification. It is better to rewrite the sentence that starts “Known techniques for SV calling are: Alignment based approaches …”. I would propose the following structure: “Known techniques for SV calling can be classified as alignment-based, assembly-based callers, and meta-callers. Alignment-based callers infer SVs by…”

Please clarify, how many times the experiment was repeated for figure 3b? Did you choose a new random interval for each experiment?

For minimap2 I suppose that it would be fair to try some of asm presets, and not include splice flag, since splice is used mostly with RNA data and doesn’t mean to use for SV discovery. Also, a newer version of minimap better handles SVs there are some large improvements. I’m also know that pbmm2 (basically minimap2) output contains supplementary alignments in order and it might identify inversions/duplication.

The GraphAligner results are far worse than what I expected for simple insertions and deletions. Can you double-check?

**Reviewer 3** (to comment on Reviewer 2’s point about the error rate of the simulated data)

I am a bit put off by their error-free simulation. They state that it is to bypass any assembly issues, but that is a pretty strong assumption to make. Given they have all of the machinery in place to do their analysis, it would be pretty trivial to crank up the error rate and then see if anything changes. Unfortunately, simulated reads are always a poor judge of performance because the errors are always so trivial. In particular, simulated data usually has uniform coverage. Real data is highly non-uniform, and its seems that interesting variants are enriched in the coverage variable regions. In short, simulated data is useful for looking at very specific dynamics but less useful when trying to determine the performance reads will likely get from the method.

**Authors’ response**

Reviewer 1

Comment 1: It is better to rewrite the sentence that starts “Known techniques for SV calling are: Alignment based approaches …”. I would propose the following structure: “Known techniques for SV calling can be classified as alignment-based, assembly-based callers, and meta-callers. Alignment-based callers infer SVs by…”

Fixed: We rewrote the respective sentence of the manuscript according to the reviewer’s suggestion.

Comment 2: Please clarify, how many times the experiment was repeated for figure 3b? Did you choose a new random interval for each experiment?

Fixed: We guess the reviewer’s comment addresses Fig. 2b and not Fig. 3b because the latter one is not related to benchmarking.

 “Did you choose a new random interval for each experiment?“

Answer: Yes, we choose a new random interval for each read.

 For answering both questions, the manuscript was extended by the following sentence: “We generate 1,000 queries for each SV-size of each SV-variant, where each query is generated from a different reference interval.” (page 7)

Comment 3: For minimap2 I suppose that it would be fair to try some of asm presets, and not include splice flag, since splice is used mostly with RNA data and doesn’t mean to use for SV discovery. Also, a newer version of minimap better handles SVs there are some large improvements. I’m also know that pbmm2 (basically minimap2) output contains supplementary alignments in order and it might identify inversions/duplication.

Fixed: We upgraded all aligners to their newest versions and repeated all experiments. The following points report about individual aligners:

 minimap2:

o We upgraded minmap2 to version: (2.25)

o The diagrams for the “asm” preset can be found in Supplementary Note 5. We decided against an inclusion of these diagrams into the main manuscript because they show no significant differences to the ones already in Fig. 2B.

o Now, the main diagram for minimap2 shows benchmarking without the splice flag.

o Although minimap2 has underwent several improvements, its general algorithmic approach hasn’t been changed since our previous benchmarking. This explains, that despite a lot of fine-tuning, there are no notable differences to the previous diagrams.

 pbmm2:

o The curves for pbmm2 can be found in in Supplementary Note 5.

o pbmm2 is a wrapper around minimap2 & samtools sort, with some additional presettings. This explains the almost identical behavior (curves) for pbmm2 and minimap2 visible in Supplementary Note 5.

Comment 4: The GraphAligner results are far worse than what I expected for simple insertions and deletions. Can you double-check?

Answer: We double checked the benchmarking for graph aligner (upgraded to version 1.0.15). The low recall rate of GraphAligner can be explained as follows:

GraphAligner gets its advantage from checking for pre-known SVs that are encoded via a graph. This graph is computed on the foundation of knowledge about already proven SV. Without this graph, GraphAligner loses all its advantages. And this is the case in our experiment. (We do denovo SV discovery.) Without a graph, GraphAligner falls back to discovering indels via the seed-chain-extend paradigm, just like many other aligners (etc. minimap2). This approach clashes with SV in many ways. For example, during the chaining, GraphAligner only combines seeds with a ‘diagonal’ distance less than 100nt ([1] –

‘Seed hit clustering’ section: “Then, two seeds in the same chain of superbubbles whose diagonal positions d1 and d2 are within a cutoff c = 100, that is, |d1 − d2| ≤ c, are connected together.” ). Indels

>100nt result in seeds outside of this 100 nt broad diagonal window. Hence, the 100 nt threshold (diagonal distance) is one reason why GraphAligner’s recall drops for indels >= ~100nt. To verify this, we manually adjusted the 100 nt threshold in GraphAligner’s code to 50 and show the resulting behaviour here:

As visible, GraphAligner’s drop in recall now happens earlier. We also tried adjusting the threshold to 200nt but could not observe significantly better recall rates. This leads us to the conclusion that there are additional filters in the chaining and extension stage that prevent alignments with indels >= 100nt. In this context, we would like to reiterate that GraphAligners behaviour reflects our argumentation in the manuscript, that alignments oppose structural variants – even in the case of insertions and deletions. This experiment was done with 2,000nt long reads and simulated sequencing errors. Note the different range on the x-axis, compared to the plots in the main text (starting from 0 instead of 50).

Reviewer 3

Comment 1: I am a bit put off by their error-free simulation. They state that it is to bypass any assembly issues, but that is a pretty strong assumption to make. […]

Fixed:

 We extended all diagrams in Fig. 2 so that they now show curves for erroneous reads as well (simulated errors). In the updated figure, the curves for error-free and errorneous reads are very close to each other. Hence our argumentation in the manuscript remains unchaged.

 There is no other benchmarking using error free reads aside Fig. 2.

 The “assembly issue” remark in our manuscript targets Fig. 5, where we work with yeast genomes. (There is no error-free simulation in Fig 5.)

The 'bypassing assembly issues' statement refers to the use of simulated reads instead of real- world reads in Fig. 5 of the original manuscript. Simulated reads offer an advantage over real- world reads as we precisely know the genome from which these reads originate, providing a ground truth. In contrast, with real-world reads, the exact genome from which the reads originate is unknown (although the assembler strives to reconstruct it, complete accuracy cannot be guaranteed), resulting in a loss of ground truth. While it is still possible to conduct an analysis using real-world reads, it involves comparing our SV caller to the assembler. Any discrepancies between the caller and the assembler are reported as errors of the SV caller, even if they may actually arise from the assembler.

To improve clarity, we modified this statement to: “We use simulated reads instead of real- world reads to establish a proper ground truth.

Comment 2: […] Given they have all of the machinery in place to do their analysis, it would be pretty trivial to crank up the error rate and then see if anything changes.

Fixed: To increase the error rate, we utilized the error profile for Oxford Nanopore reads provided by SURVIVOR (NA12878_nano_error_profile) and repeated the benchmarking analysis of Figure 5. The resulting curves can be found in Supplementary Note 6. For smaller SVs (<10nt), the high error rate in Oxford Nanopore reads has moderate impact to recall and accuracy. However, for larger SVs, the presence of read errors introduces increasing noise in the adjacency matrix, resulting in decreased recall and accuracy rates.

Comment 3: Unfortunately, simulated reads are always a poor judge of performance because the errors are always so trivial. In particular, simulated data usually has uniform coverage. Real data is highly non-uniform, and its seems that interesting variants are enriched in the coverage variable regions. In short, simulated data is useful for looking at very specific dynamics but less useful when trying to determine the performance reads will likely get from the method.

Fixed: Figure 5 now additionally comprises curves for “real-world reads” (taken from [2]). These additional curves show that our approach can cope with real world data as well. As the reviewer states, real word reads normally express a less even coverage that simulated reads. For addressing this issue, we extend our approach by the computation of a coverage histogram. This histogram is then used during the scoring of SV calls. The details have been added to the methods section.

References

[1] M. Rautiainen and T. Marschall, “GraphAligner: rapid and versatile sequence-to-graph alignment,”

Genome Biol, vol. 21, no. 1, p. 253, Sep. 2020, doi: 10.1186/s13059-020-02157-2.

[2] F. Giordano et al., “De novo yeast genome assemblies from MinION, PacBio and MiSeq platforms,”

Scientific Reports, vol. 7, no. 1, p. 3935, Jun. 2017, doi: 10.1038/s41598-017-03996-z.

**3^rd^ round**

**Reviewer 2**

I am satisfied with the revisions made to this manuscript. No further comments or suggestions.
